# Data on the diets of Salish Sea harbour seals from DNA metabarcoding

**DOI:** 10.1038/s41597-022-01152-5

**Published:** 2022-03-02

**Authors:** Austen C. Thomas, Bruce Deagle, Chad Nordstrom, Sheena Majewski, Benjamin W. Nelson, Alejandro Acevedo-Gutiérrez, Steven Jeffries, Jed Moore, Amelia Louden, Hassen Allegue, Scott Pearson, Michael Schmidt, Andrew W. Trites

**Affiliations:** 1 Molecular Division, Smith-Root Inc., 16603 NE 50th Avenue, Vancouver, WA 98686 USA; 2 University of British Columbia, Institute for the Oceans and Fisheries, 247-2202 Main Mall, Vancouver, BC V6T 1Z4 British Columbia Canada; 3 Australian National Fish Collection, CSIRO National Research Collections Australia, Hobart, TAS Australia; 4 Australian Antarctic Division, Channel Highway, Kingston, TAS Australia; 5 Pacific Biological Station, 3190 Hammond Bay Road, Fisheries and Oceans Canada, Nanaimo, BC V9T 6N7 Canada; 6 Department of Biology, Western Washington University, Bellingham, WA 98225 USA; 7 Washington Department of Fish and Wildlife, Olympia, WA 98501 USA; 8 Nisqually Indian Tribe, Olympia, WA 98513 USA; 9 Université du Québec à Montréal, Département des Sciences Biologiques, 141 Avenue du Président-Kennedy, Montréal, H2X 1Y4 QC Canada; 10 Long Live the Kings, 1326 5th Avenue, 450, Seattle, Washington 98101 USA

**Keywords:** Animal behaviour, Marine biology, Ecological modelling, Molecular ecology

## Abstract

Marine trophic ecology data are in high demand as natural resource agencies increasingly adopt ecosystem-based management strategies that account for complex species interactions. Harbour seal (*Phoca vitulina*) diet data are of particular interest because the species is an abundant predator in the northeast Pacific Ocean and Salish Sea ecosystem that consumes Pacific salmon (*Oncorhynchus spp*.). A multi-agency effort was therefore undertaken to produce harbour seal diet data on an ecosystem scale using, 1) a standardized set of scat collection and analysis methods, and 2) a newly developed DNA metabarcoding diet analysis technique designed to identify prey species and quantify their relative proportions in seal diets. The DNA-based dataset described herein contains records from 4,625 harbour seal scats representing 52 haulout sites, 7 years, 12 calendar months, and a total of 11,641 prey identifications. Prey morphological hard parts analyses were conducted alongside, resulting in corresponding hard parts data for 92% of the scat DNA samples. A custom-built prey DNA sequence database containing 201 species (192 fishes, 9 cephalopods) is also provided.

## Abstract (summary)

**Table:** 

Measurement(s)	diet
Technology Type(s)	DNA metabarcoding diet analysis
Sample Characteristic - Organism	Phoca vitulina

Machine-accessible metadata file describing the reported data: 10.6084/m9.figshare.19077809

## Background & Summary

In recent years the fisheries and ecosystem modelling communities have expressed increased interest in harbour seal (*Phoca vitulina*) diet information to help inform prey consumption estimates and ecosystem models^1–3^. Harbour seals are a common pinniped found in the northeast Pacific Ocean and are particularly abundant in the inland marine waters of southern British Columbia (Canada) and Washington state (USA) – an area defined as the Salish Sea^4,5^. Growth in the population of seals since their protection in 1972 has led to concerns about their potential predatory impacts on fishes of conservation concern such as Pacific salmon (*Oncorhynchus* spp.)^6^. In addition to being known salmon predators, the inverse population trend between seals and salmon species has led to speculation that seals may be a causal factor influencing salmon populations^7,8^. More specifically, the marine survival of Chinook (*O. tshawytscha*), coho (*O. kisutch*), and steelhead (*O. mykiss*) salmon in the region decreased dramatically during the same time period when harbour seals grew exponentially^7,9,10^. However, such correlative evidence is generally not sufficient to mandate management actions. Additional data, such as detailed predator diet information, is needed to establish a potential ecological link between seals and fish populations^2,11^.

Although harbour seal diet in the region has been studied for nearly a century^12^, methodological limitations have prevented accurate quantification of the salmon proportion of seal diet. Previous studies relied primarily on morphological identification of hard remains (e.g., bones) in stomachs or scat samples, and diet was summarized based on the proportion of samples containing bones of certain prey species^13,14^. Unfortunately, the majority of salmon bones in scats cannot easily be identified to the species level, and simple prey occurrence data are of limited use for quantitative analyses of predation. Such estimates generally require (as an input) the predator population diet fraction comprised of a particular prey species^1,2^. A new harbour seal diet method was therefore needed to meet the data requirements of regional modelling efforts.

In 2011, collaborating researchers at the University of British Columbia (UBC) and the Australian Antarctic Division undertook an effort to create a new diet analysis method capable of providing both the salmon species and the proportional biomass of prey contained in harbour seal scat samples. At the time, analysis of predator diet using DNA metabarcoding methods was a new approach that offered the potential to provide both the seal prey species and the relative proportions of prey in scat samples^15^. High-resolution taxonomic data can be achieved with DNA metabarcoding using massively parallel sequencing of short (e.g., 200 bp), diagnostic genetic markers amplified from scat samples^15,16^. Sequences are compared to a database containing known DNA barcodes of potential prey species, and the proportions of DNA sequences assigned to different prey are used as an index of proportional biomass composition for each scat sample^16^. The product of the described R&D effort was a DNA metabarcoding diet analysis method for harbour seals that has since been applied to thousands of wild harbour seal scat samples collected throughout the Salish Sea^8,17^.

Between the years of 2011 and 2019, over four-thousand regional harbour seal scat samples were collected and processed using our standardized DNA metabarcoding diet analysis method. This is the product of a collaborative transboundary research effort including two universities (University of British Columbia, Western Washington University), three government agencies (Washington Department of Fish and Wildlife, Fisheries and Oceans Canada, Australian Antarctic Division), a native American tribe (Nisqually Indian Tribe), a non-profit organization (Long Live the Kings) and private corporation (Smith-Root Inc.). Subsets of the data have been used to address specific questions with respect to pinniped predation on particular prey species or taxa^1,2,8^. Further, the diet analysis method produces proportional data for all prey species amplified by the semi-universal PCR primers; therefore, these data products are also useful for a broad range of ecological questions and modelling exercises that extend beyond the previous taxon-specific inquiries.

Our objective in publishing this dataset is to make these valuable trophic ecology data broadly available to the fisheries and ecosystem modelling communities. The core of the dataset is a single spreadsheet containing data from 4,625 harbour seal scats representing 52 haulout sites, 7 years, 12 calendar months, and totaling 11,641 prey identifications. We hope that by making these data publicly available they will help facilitate an open and free exchange of ideas regarding harbour seal trophic ecology in the Salish Sea.

## Methods

### Scat sample collection and preparation

**Fig. 1:**
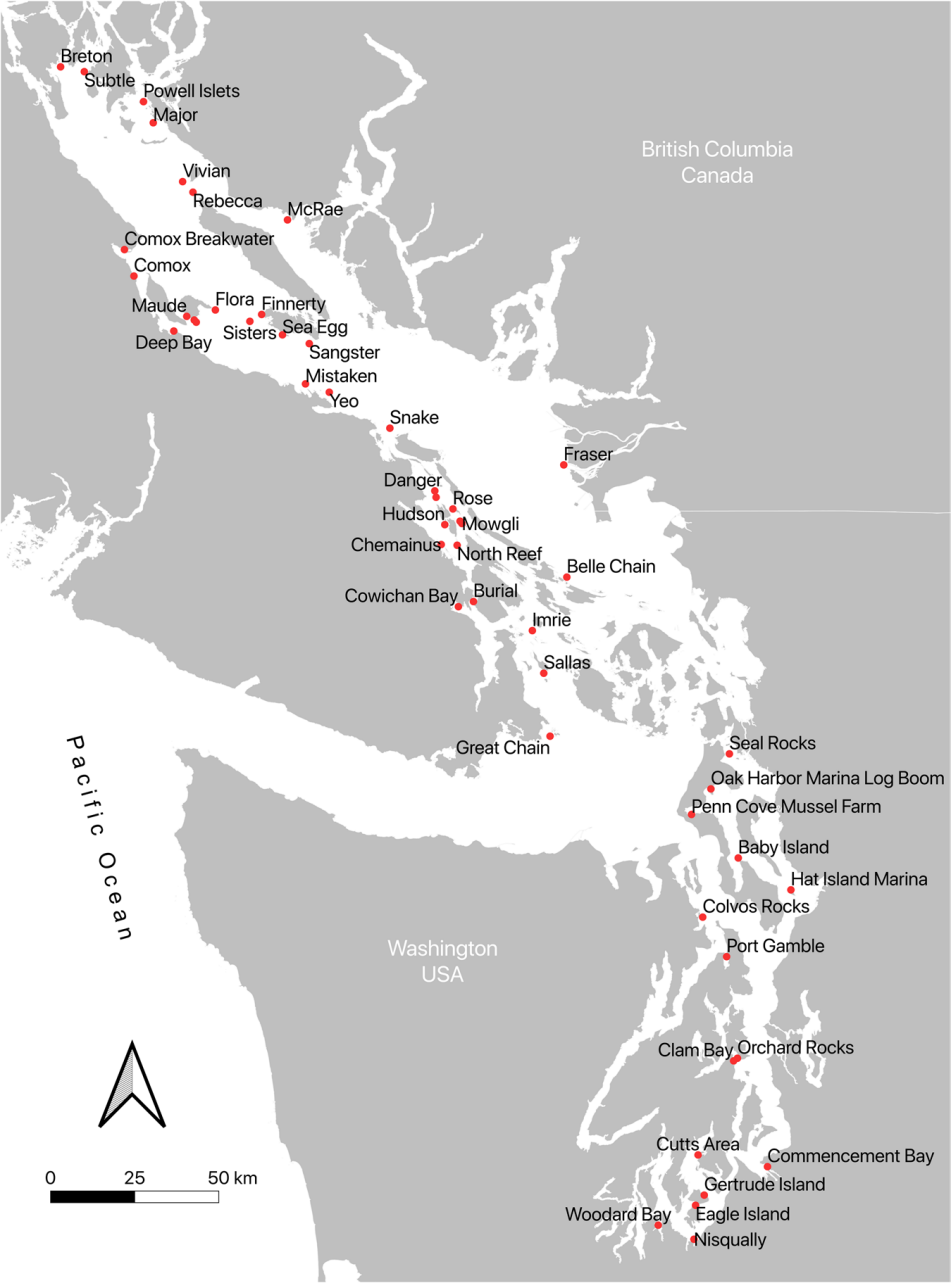
The 52 harbour seal scat collection sites in the Salish Sea represented in this dataset.

At known harbour seal haulout sites individual scat samples were collected using a standardized protocol (Fig. 1). Disposable wooden tongue depressors were used to transfer deposited scats into 500 ml single-use jars or zip-style bags lined with 126 µm nylon mesh paint strainers^18^. Samples were either preserved immediately in the field by adding 300 ml 95% ethanol to the collection jar, or were taken to the lab and frozen at −20 °C within 6 hours of collection^19^. Later, samples were thawed and filled with ethanol before being manually homogenized with a disposable wooden depressor inside the paint strainer to separate the scat matrix material from hard prey remains (e.g. bones, cephalopod beaks). The paint strainer containing prey hard parts was then removed from the jar leaving behind the ethanol preserved scat matrix for genetic analysis^20^. The paint strainer containing prey hard parts was refrozen for subsequent parallel morphological prey ID.

### Molecular laboratory processing

Scat matrix samples were subsampled (approximately 20 mg), centrifuged and dried to remove ethanol prior to DNA extraction. DNA was extracted from scat with the QIAGEN QIAamp DNA Stool Mini Kit according to the manufacturer’s protocols. For additional details on the extraction process see Deagle *et al*.^21^ and Thomas *et al*.^20^.

The metabarcoding marker we used to quantify fish and cephalopod proportions was a 16S mDNA fragment (~260 bp) previously described in Deagle *et al*.^15^ for pinniped scat analysis. We used the combined Chord/Ceph primer sets: Chord_16S_F (GATCGAGAAGACCCTRTGGAGCT), Chord_16S_R (GGATTGCGCTGTTATCCCT), and Ceph_16S_F (GACGAGAAGACCCTAWTGAGCT), Ceph_16S_R (AAATTACGCTGTTATCCCT). This multiplex PCR reaction is designed to amplify both chordate and cephalopod prey species DNA. A blocking oligonucleotide was included in the all 16S PCRs to limit amplification of seal DNA^22^. The oligonucleotide (32 bp: ATGGAGCTTTAATTAACTAACTCAACAGAGCA-C3) matches harbour seal sequence (GenBank Accession AM181032) and was modified with a C3 spacer so it is non-extendable during PCR^22^.

A secondary metabarcoding marker was used in a separate PCR reaction to quantity the salmon portion of seal diet, because the primary 16S marker was unable to reliably differentiate between coho and steelhead DNA sequences. This marker was a COI “minibarcode” specifically for salmonids within the standard COI barcoding region: Sal_COI_F (CTCTATTTAGTATTTGGTGCCTGAG), Sal_COI_R (GAGTCAGAAGCTTATGTTRTTTATTCG). The COI amplicons were sequenced alongside 16S such that the overall salmonid fraction of the diet was quantified by 16S, and the salmon species proportions within that fraction were quantified by COI.

To take full advantage of sequencing throughput, we used a two-stage labeling scheme to identify individual samples that involved both PCR primer tags and labeled MiSeq adapter sequences. The open source software package EDITTAG was used to create 96 primer sets each with a unique 10 bp primer tag and an edit distance of 5; meaning that to mistake one sample’s sequences for another, 5 insertions, substitutions or deletions would have to occur^23^.

All PCR amplifications were performed in 20 μl volumes using the Multiplex PCR Kit (QIAGEN). Reactions contained 10 μl (0.5 X) master mix, 0.25 μM of each primer, 2.5 μM blocking oligonucleotide and 2 μl template DNA. Thermal cycling conditions were: 95 °C for 15 min followed by 34 cycles of: 94 °C for 30 s, 57 °C for 90 s, and 72 °C for 60 s.

Amplicons from 96 individually labeled samples were pooled by running all samples on 1.5% agarose gels, and the luminosity of each sample’s PCR product was quantified using Image Studio Lite (Version 3.1). To combine all samples in roughly equal proportion (normalization), we calculated the fraction of each sample’s PCR product added to the pool based on the luminosity value relative to the brightest band. After 2013, amplicon normalization was performed using SequalPrep™ Normalization Plate Kits, 96-well.

Sequencing libraries were prepared from pools of 96 samples using an Illumina TruSeq DNA sample prep kit which ligated uniquely labeled adapter sequences to each pool. Libraries were then pooled and DNA sequencing was performed on Illumina MiSeq using the MiSeq Reagent Kit v2 (300 cycle) for SE 300 bp reads. Samples were sequenced on multiple different runs as part of the larger study; however, typically between 4 and 6 libraries (each a pool of 96 individually identifiable samples) were sequenced on a single MiSeq run.

### Bioinformatics

To assign DNA sequences to a fish or cephalopod species, we created a custom BLAST reference database of 16S sequences by an iterative process. First, using a list of the fish species of Puget Sound, we searched Genbank for the 16S sequence fragment of all fishes known to occur in the region (71 fish families 230 species)^24,25^. Reference sequences for each prey species were included in the database if the entire fragment was available, and preference was given to sequences of voucher specimens. When the database was first generated (November, 2012) Genbank contained 16S sequences for 192 of the 230 fish species in the region, and the remaining 38 species were mostly uncommon species unlikely to occur in seal diets. Following a similar procedure, we added to this database sequences for all of the regional cephalopods for which 16S data were available (7 squid species, 2 octopus species). A separate reference database was generated for the COI salmon marker containing Genbank sequences for the nine salmonid species known to occur regionally: *Oncorhynchus gorbuscha* (Pink Salmon), *Oncorhynchus keta* (Chum Salmon), *Oncorhynchus kisutch* (Coho Salmon), *Oncorhynchus mykiss* (Steelhead), *Oncorhynchus nerka* (Sockeye Salmon), *Oncorhynchus tshawytscha* (Chinook Salmon), *Oncorhynchus clarkii* (Cutthroat Trout), *Salmo salar* (Atlantic Salmon), *Salvelinus malma* (Dolly Varden)^24^.

To determine if some species in the database cannot be distinguished from each other at 16S (i.e. have identical sequences in the reference database) a distance matrix was performed on the complete database using the DistanceMatrix function in the R package DECIPHER^26^. Species with identical sequences were identified as having a distance of “0.00”. In some cases, one haplotype for a species was identical to another species but other haplotypes were not. When two species’ sequences were identical, we ultimately reported both species in the prey_ID field.

Sequences were automatically sorted (MiSeq post processing) by amplicon pool using the indexed TruSeqTM adapter sequences. FASTQ sequence files for each library were imported into MacQIIME (version 1.9.1-20150604) for demultiplexing and sequence assignment to species^27^. For a sequence to be assigned to a sample, it had to match the full forward and reverse primer sequences and match the 10 bp primer tag for that sample (allowing for up to 2 mismatches in either primers or tag sequence).

Next, we clustered the DNA sequences that were assigned to scat or tissue samples with USEARCH (similarity threshold = 0.99; minimum cluster size = 3; de novo chimera detection), and entered a representative sequence from each cluster into a GenBank nucleotide BLAST search^28,29^. If the top matching species for any cluster was not included in the existing database (or the sequence differed indicating haplotype variation), we put the top matching entry in the reference database. We repeated this procedure with every new batch of sequence data to minimize the potential for incorrect species assignment or prey species exclusion. This process was conducted for both the 16S and COI reference databases with each new batch of samples.

For all DNA sequences successfully assigned to a sample, a BLAST search was performed against our custom 16S or COI reference databases. A sequence was assigned to a species based on the best match in the database (threshold BLASTN e-value < 1e-20 and a minimum identity of 0.9), and the proportions of each species’ sequences were quantified by individual sample after excluding harbour seal sequences or any identified contaminants^27^. Samples were excluded from subsequent analysis if they contained <10 identified prey DNA sequences (given the current costs of DNA sequencing, a higher threshold is now advisable). Harbour seal DNA diet percentages for individual scats were then calculated using the Relative Read Abundance (RRA) calculation commonly used in metabarcoding studies (Box 1)^16^. The RRA formula was used to calculate the “DNA_diet_percent” field in data record “Harbour_Seal_DNA_Diet_Data.csv”.

Box 1 (From Deagle *et al*.^16^). Metrics used to summarise sequence data in dietary studies. ***Occurrence Data***

Frequency of occurrence (FOO) is the number of samples that contain a given food item, most often expressed as a percent (%*FOO*). Percent of occurrence (*POO*) is simply %*FOO* rescaled so that the sum across all food items is 100%. Weighted percent of occurrence (*wPOO*) is similar to *POO*, but rather than giving equal weight to all occurrences, this metric weights each occurrence according to the number of food items in the sample (e.g., if a sample contains 5 food items, each will be given weight 1/5). Mathematical expressions are as follows:

$${\rm{ \% }}FO{O}_{i}=\frac{1}{S}\mathop{\sum }\limits_{k=1}^{S}{I}_{i,k}\times 100 \% $$

$$PO{O}_{i}=\frac{{\sum }_{k=1}^{S}{I}_{i,k}}{{\sum }_{i=1}^{T}{\sum }_{k=1}^{S}{I}_{i,k}}$$

$$wPO{O}_{i}=\frac{1}{S}\mathop{\sum }\limits_{k=1}^{S}\frac{{I}_{i,k}}{{\sum }_{i=1}^{T}{I}_{i,k}}$$

where *T* is the number of food items (taxa), *S* is the number of samples, and *I* is an indicator function such that *I*_*i,k*_ = 1 if food item *i* is present in sample *k*, and 0 if not.

Many metabarcoding diet studies make use of both %FOO and POO e.g.^42^. POO provides a convenient view since each food taxon contributes a percentage of total diet (unlike %FOO which does not sum to 100%). In POO summaries samples with a high number of food taxa have a stronger influence, whereas in wPOO each sample is weighted equally (i.e. lower weighting to food taxa in a mixed meal) and this may be more biologically realistic. wPOO is the same as split-sample frequency of occurence; see^43^ and references within.

***Read Abundance Data***

Using the sequence counts, relative read abundance (*RRA*_*i*_) for food item *i* is calculated as:

$${RRA}_{i}=\frac{1}{S}\mathop{\sum }\limits_{k=1}^{S}\frac{{n}_{i,k}}{{\sum }_{i=1}^{T}{n}_{i,k}}\times 100 \% $$

where *n*_*i,k*_ is the number of sequences of food item *i* in sample *k*.

### Prey hard parts analysis

Extraction and identification of hard structures from harbour seal scats was conducted by three different analysts. We used the “all structures” approach to identify harbor seal prey contained in individual scat samples, which make our results comparable to similar studies previously conducted in the region^8,13,14^. Prey “hard parts” retained in paint strainers were cleaned of debris using either a conventional washing machine or nested sieves. All diagnostic prey hard parts were identified to the lowest possible taxon using a dissecting microscope and reference fish bones from Washington and British Columbia, in addition to published keys for fish bones and cephalopod beaks^30–33^. Samples containing prey hard parts identifiable only to the family level (e.g., Clupeidae), and bones identifiable to the species level of the same family (e.g., Pacific herring, Clupea pallasii) were both tallied.

In previous studies of harbour seal impacts to juvenile salmonids in the Salish Sea^1,2,8,34^, these diagnostic hard structures (e.g., otoliths, bones) were combined with DNA extracted from each scat sample to estimate the proportion of juvenile and adult salmon (by species) in the seal diet. This approach (see: Thomas *et al*.^8^) integrates separate analyses of hard parts and DNA through an algorithm that apportions the salmonid DNA component in each sample to a “juvenile” or “adult” classification. The decision algorithm is based on the co-occurrence of age-classified salmon bones and salmon DNA in samples, and (when bones were not present but salmon DNA was detected) on known seasonal life-history information. For example, an individual scat sample found to contain 5% Chinook salmon, and a 1:1 ratio of juvenile to adult salmon bones, would be disaggregated into a final classification of 2.5% juvenile Chinook salmon and 2.5% adult Chinook salmon. For individual scat samples that do not contain diagnostic hard structures, the ratio of juveniles to adults in that sample would rely on the ratio of hard parts pooled for the collection month. If no hard structures are available for the collection month-which only occurred for 7% of samples in Thomas *et al*.^8^ a seasonal classification would then be applied to the sample (spring = juvenile, fall = adult). The classification of hard parts as “juvenile” or “adult” was performed by taxonomic experts who differentiated samples visually, and/or according to otolith or vertebral measurements (e.g., Nelson *et al*.^34^).

## Data Records

**Fig. 2:**
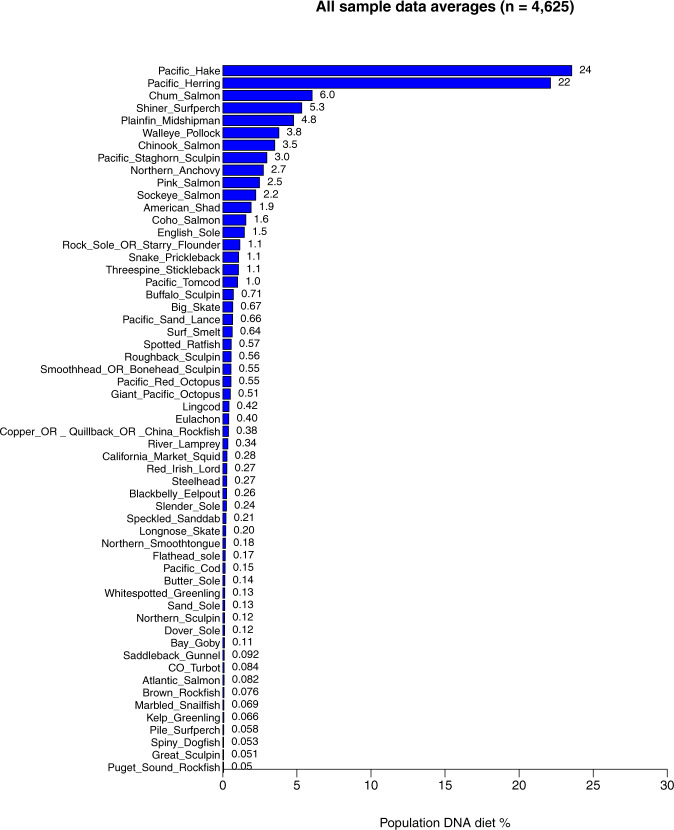
Harbour seal DNA diet percent averages (RRA) for all of the Salish Sea samples combined. Please note that considerable diet variability exists between haulout sites and regions in the Salish Sea.

A summary figure depicting the average diet of the complete Salish Sea harbour seal dataset comprised of 4,625 scat samples is available in Fig. 2.

The following files are available at figshare^35^.

### Harbour_seal_dna_diet_data.csv

This is the primary dataset that contains scat sample composition data for 4,625 harbor seal scats. Field names and descriptions are outlined in Table 1. Multiple entries exist for each sample ID, with each entry representing an identified prey species and its proportional composition within the sample.

**Table 1:** Field names and descriptions of the data contained in “Harbour_Seal_DNA_Diet_Data.csv”.

Field	Example	Description
sample_ID	Pv16.E090	Project assigned sample ID
site	Belle Chain	Collection site name
Lat	48.8323	Site latitude
Lon	−123.1984	Site longitude
day	19	Scat colleciton day
month	9	Scat colleciton month
year	2016	Scat colleciton year
predator	Harbour seal	Predator species
prey_code	ONCTSH	Six-letter prey species code
prey_ID	Oncorhynchus_tshawytscha	Prey species taxonomic ID
common_name	Chinook_Salmon	Prey species common name
count.16S	184	16S DNA sequence count assigned to that prey species
count.COI	4963	COI DNA sequence count assigned to that prey species (only salmon)
DNA_diet_fraction	0.201164835	Fractional component of scat DNA sequences assigned to prey species
DNA_diet_percent	20.11648352	Percentage of scat DNA sequences assigned to prey species
Lab	DFO	The molecular laboratory where work was conducted
Bioinformatician	Nordstrom	The analyst responsible for generating diet data summaries
File	dna_new_BC_2015-16-17.csv	The output project file used

Notes for Harbour_Seal_DNA_Diet_Data.csv:

The sample ID nomenclature varied between projects and we present here the IDs used by the respective projects and institutions. This presentation allows for links to drawn to other parallel projects or datasets using the common sample IDs.

Similarly, the collection location names were assigned by the separate projects and do not necessarily represent specific cartographic locations. Therefore, the Latitude and Longitude are provided in decimal degree format for each sampling location.

Database duplicates: When the prey ID sequence in the database was a perfect match for another prey species sequence, both prey IDs are provided separated by “OR” in the entry. For example, “Copper_OR _ Quillback_OR _China_Rockfish”. The same approach was used for the taxonomic names of the prey species.

DNA_diet_percent is the product of the RRA calculation for each prey species and can be used as an index of proportional prey consumption (see methods).

### Harbour_seal_hardparts_diet_data.csv

**Fig. 3:**
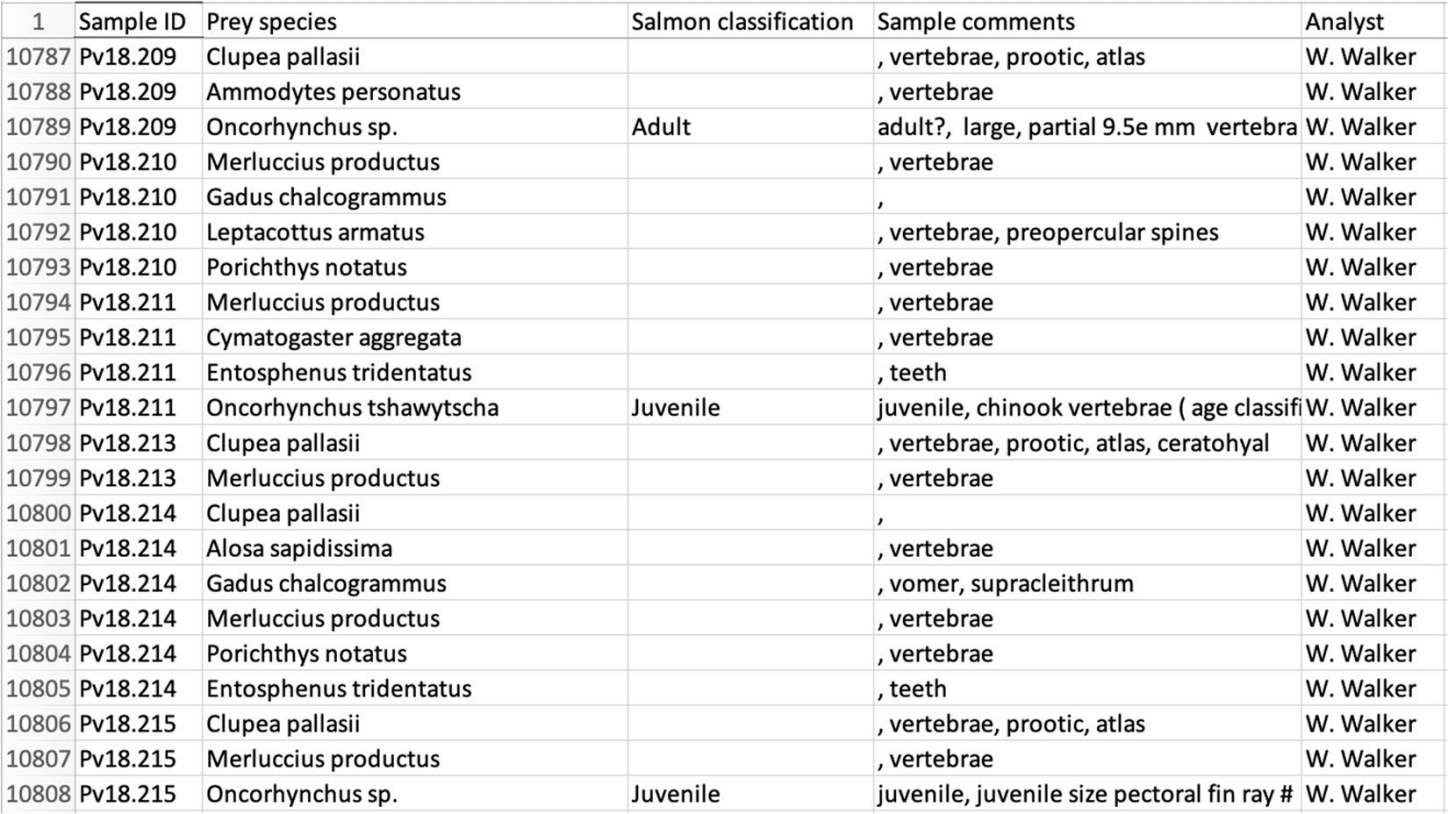
A screen capture of the harbour seal hard parts diet data file, illustrating example data.

These are the results of the morphological prey hard parts ID of collected scat samples, with corresponding sample IDs to those in Harbour_Seal_DNA_Diet_Data.csv (Fig. 3). These data can be used to make direct comparisons between the traditional seal diet analysis technique (prey hard parts analysis) and the newly developed diet characterization method (DNA metabarcoding diet analysis).

Notes for Harbour_Seal_Hardparts_Diet_Data.csv:

The.csv file contains data in five fields:

1. Sample ID – the scat sample ID that corresponds to “sample ID” in the DNA dataset.
2. Prey Species – the prey species scientific name.
3. Salmon classification – Binary age classification for salmon structures as noted by the analyst.
4. Sample comments – Additional comments about the specific prey hard structure.
5. Analyst – The name of the prey hard parts identification analyst.

Because the prey hard parts analysis was conducted by three different analysists without an existing standardization protocol, the reported results varied slightly throughout the dataset. Here we attempted to preserve as much of information provided by the analyst as possible in the “Sample comments” field.

Further, given the intense intertest in salmon predation by harbour seals and the large magnitude differences in consumption estimates depending on prey life stage, we have included the binary salmon age classification (Juvenile, Adult). We should acknowledge, however, that seals consume salmon prey in a wide range of age/size classes, and this binary scheme is an oversimplification.

### Prey_database.txt

**Fig. 4:**
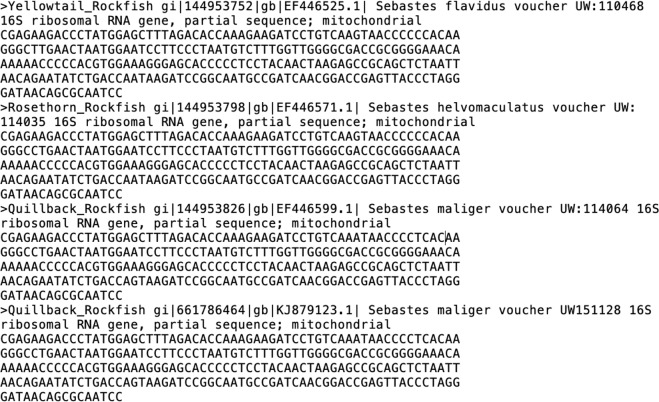
A screen capture example of the prey database FASTA file.

The prey reference sequence database (Fig. 4) is a product of the iterative process of adding prey sequences based on the representative sequences of clustered MiSeq data from each successive scat sequencing run. The database started with a Genbank search for 16S sequences using a list of local fish species, and then the clustering processes for each sequencing run added additional species or haplotypes to the database over time.

### Notes for prey_database.txt

This version of the database does not contain identified contaminants or predators other than harbour seal that were inadvertently collected.

### Prey_species_distance_matrix.xlsx

This distance matrix was used to determine when the reference sequences of two prey species in the database were identical. Both prey species names are given in the Prey_ID field of the diet dataset in those occurrences.

Notes for Prey_Species_Distance_Matrix.xlsx:

Multiple sequence entries exist for some prey species as a byproduct of the iterative sequence entry process. In some cases, one haplotype for a species was identical to another species but other haplotypes were not.

### QIIME_mapping_files.tgz

A tar archive of the QIIME mapping files (.txt) needed for demultiplexing the.fastq files in the NCBI SRA database (Online-only Table 1)^36^. The mapping file names in the tar archive match those of the associated.fastq files. Each mapping file contains the F and R primer sequences along with the unique primer tag for each of the 96 scat samples listed in the sequencing plate.

## Technical Validation

In parallel to the field sample collection effort, we conducted a series of studies to evaluate the quantitative capabilities of DNA metabarcoding diet analysis for seal diet estimation. Specifically, we wanted to determine if prey DNA sequence percentages recovered from scats (RRA) accurately reflect the proportional biomass of the prey consumed. This work involved a series of experiments including: a captive seal feeding study with known diets, the development of food tissue control materials (homogenized fish tissue) to produce correction factors, and computer simulations to compare RRA to alternative diet indices such as weighted percent of occurrence. Here we will briefly describe those studies and the principal conclusions drawn from each. Lastly, we will outline why we have chosen to report RRA as the principal diet index for this dataset.

Ideally, RRA would be a direct reflection of the proportional biomass of the prey species consumed – i.e. a 1:1 relationship would exist between metabarcoding DNA sequence proportion and diet biomass proportion. However, a large number of potential factors could skew this relationship, including variability in mDNA density (number of mitochondria per gram of tissue) between different prey species, technical biases such as preferential primer binding during PCR caused by primer mismatches, and the many bioinformatic processes that may select for one prey species’ sequences over another. For this reason, our first validation study was an evaluation of the factors that influence the relationship between diet biomass proportion and RRA in a captive seal feeding study wherein aquarium animals were fed a known diet of fixed biomass proportions of three prey species^37^.

In that study using the Ion Torrent PGM sequencer, we found that DNA sequence quality filtering, direction of sequencing (F vs R), and our chosen minimum read length, all largely impacted the proportions of prey sequences ultimately resulting from RRA analysis^37^. Furthermore, we detected interactive effects between biasing factors such as variable effects of quality filtering depending on the primer tags used to identify sequences from unique samples. However, despite these effects we found that replicate samples of a common diet produced largely consistent DNA sequence percentages when technical factors were held constant. This consistency implied that biasing factors could potentially be corrected using a set of standards sequenced alongside scat samples, such as homogenized mock communities of known composition (i.e. prey fish tissue mixes).

The next study therefore attempted to correct for biasing factors by sequencing a prey fish tissue mixture that matched the diet biomass proportions of captive seals, allowing us to calculate and apply Tissue Correction Factors (TCFs) to the scat DNA sequence counts^20^. When applied to seal scat DNA, TCFs substantially improved the relationship between RRA and prey biomass proportion for all prey species. We also surmised that while the TCFs account for technical biases (e.g. quality filtering, differential primer binding) and mDNA density variability, they did not account for any potential effects of differential prey species digestion (e.g., one species being more fully digested than another in the seal’s gastrointestinal tract). The study design of the experiment allowed us to quantify the bias introduced by differential prey digestion and determine in that case the magnitude of the required Digestion Correction Factor (DCF) for each species. It is unrealistic to experimentally determine the DCF for all prey when hundreds of prey species are possible, so we explored prey characteristics (proximate composition analysis) that could be used as a proxy for prey digestion. We found that the percent lipid of prey species closely predicted the DCF, implying that highly accurate diet biomass estimates could be obtained by applying both TCFs and lipid-based DCF correction factors. The correction factor design of this experiment however relied on *a priori* knowledge of the seal diets; therefore, a more generalizable approach was needed that could be applied to samples of unknown composition.

Using the lessons learned from the captive feeding studies, we thus created a novel correction factor approach for samples of unknown composition by generating 50/50 biomass percentage mixtures of variable potential prey fish combined with a “control” fish that was held constant in all mixtures^38^. By holding one of the two prey constant in all 50/50 mixtures, we were able to detect biases in the “test” prey relative to the control species and to calculate Relative Correction Factors (RCFs) that could be applied to DNA sequence counts from samples of unknown biomass composition (i.e. seal scat samples). We then built a prey library of the 50/50 mixtures and sequenced them alongside seal scat samples, calculating RCFs for prey species in the library and applying them to scat samples to determine the magnitude of corrective effect. Similar to previous results, RCFs were highly consistent between sequencing replicates, and RCF values indicated that there is some phylogenetic structure to the magnitude of bias (active swimming fishes were overestimated relative to more sedentary species, likely reflecting mDNA density). The effects of RCF correction were most pronounced on individual samples, although when samples were averaged together (as is common practice when calculating population level diet summaries) the effect of RCF correction were less impactful. We ultimately concluded that RCF correction is only a worthwhile endeavor when a high degree of diet biomass accuracy is needed for a single sample, or when generating population diet summaries from a small number of diet samples.

Despite the known biases, researchers have consistently concluded that RRA is a semi-quantitative tool even without the application of correction factors. When the diet proportion of a prey species increases, there is a corresponding increase in the relative number of DNA sequences for that prey species in diet samples. So that begs the question, are uncorrected “semi- quantitative” RRA estimates good enough? One way to answer this question is to compare the accuracy of the RRA method to other competing methodological alternatives. DNA metabarcoding RRA may be subject to known biasing factors, but if it outperforms alternative diet metrics in terms of taxonomic resolution and biomass estimate accuracy, then it may be the current best option for pinniped diet analysis.

The most common practice for pinniped diet studies is to treat detections of prey species in diet samples as presence/absence data (i.e., occurrences). Percent of occurrence (POO) tables can be generated for groups of samples using such data, or when proportions must sum to 1 (as is needed for bioenergetics modelling) a weighted percent of occurrence (wPOO) can be calculated (Box 1). The latter has also been called Split Sample Frequency of Occurrence (SSFO). Occurrence-based indices however are prone to overestimating prey eaten in low proportion and underestimating prey eaten in high proportions. In a metanalysis of DNA metabarcoding diet studies, Deagle *et al*.^16^ compared multiple diet indices using an *in silico* simulation experiment. Diet datasets were simulated at multiple sample sizes and the proportional composition was calculated using RRA and occurrence indices, then compared to the theoretical true diet using Bray–Curtis dissimilarity. They found that occurrence indices produce consistent diet estimates (relatively precise) but result in a less accurate reflection of the true diet by comparison to RRA. RRA produced a more accurate estimate than POO even when the simulated level of bias was extreme (20X), suggesting that the quantitative signature of RRA is important for generating accurate diet estimates despite the presence of biases.

**Fig. 5:**
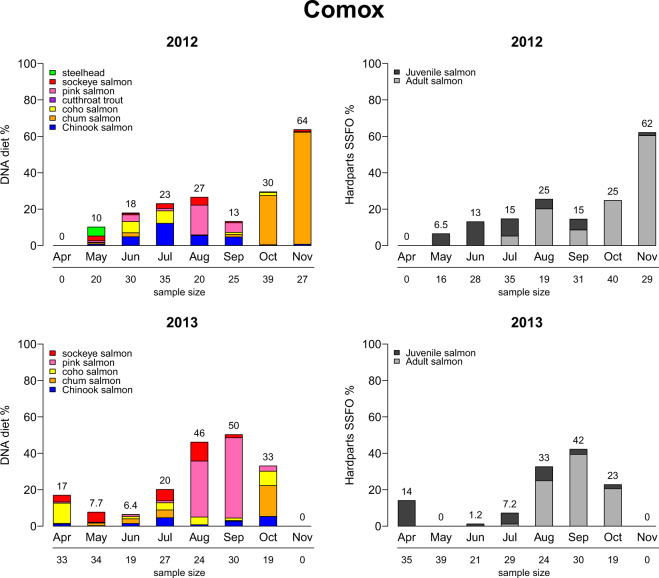
Figure B-2 from Thomas^17^, illustrating the general agreement between scat DNA RRA (Left) and prey hard parts analysis using wPOO (Hard parts SSFO%) (Right) from the same set of scat samples. Data shown are the salmon component of harbour seal diet from the Comox collection site over two years: 2012 (Top), 2013 (Bottom).

Comparing DNA metabarcoding RRA to non-DNA based methods with a proven history of use (e.g., scat morphological hard parts analysis) is another means of assessment. In an extensive methods comparison between scat DNA and hard parts analysis, Thomas (2015)^17^ found a high degree of agreement between the two methods in the proportions of salmon estimated in seal diets (Fig. 5), in addition to other diet species (Online-only Table 2). Furthermore, in a recent report^39^, pinniped diet experts Dominic Tollit and Ruth Joy compared DNA metabarcoding RRA (Termed “DNA-Fixed” in report) to the current best practices method (hard parts biomass reconstruction – “BR-Fixed”) and found “near identical” diet contributions between methods (see Supplementary Information for full report, posted with permission). This implies that DNA metabarcoding RRA provides not only better taxonomic resolution than hard parts, but also produces comparable diet proportions to the current best practices method while requiring substantially less labor. A copy of the report is available in Supplementary Information.

Given the strengths of RRA for proportional biomass estimation in the absence of correction factors, the cost/labor involved in generating a complete prey library for RCF correction, and fact that harbour seal prey are minimally biased with our 16S marker, we have chosen to present uncorrected RRA as the proportional diet index in the current dataset. Caution should therefore be exercised when generating diet summaries using small numbers of samples (e.g.< 70 per stratum), as is considered best practice with other diet indices^40^. Future efforts may be made to build a 50/50 RCF prey library for harbour seals in the Salish Sea region, in which case the diet database presented here could be RCF corrected for improved sample proportional biomass accuracy. It should also be noted that our methods evaluations did not account for other potential sources of bias (e.g., sampling bias introduced during scat collection, selective influence of blocking oligos, secondary prey consumption, reliance on Genbank for reference database sequences, etc.). Those issues are worth investigating further in future methods refinement.

## Usage Notes

As stated previously, we encourage users of these data to follow statistical best-practices when creating diet summaries from this dataset. Ample literature is available on the topic of appropriate size for predator diet studies^40,41^.

## Supplementary information

Supplementary Information

## Data Availability

The bioinformatic code used to process scat DNA sequence data are available in the “Example Bioinformatic Files.zip” folder on figshare along with example data^35^. Sample demultiplexing and sequence taxonomy assignment steps were performed using MacQIIME (version: 1.9.1-20150604) and the code used for these steps is in folder “1_Qiime_processing”. Example output files from MacQIIME processing steps containing 16S and COI sequence taxonomy assignments are in the folder “2_Qiime_output_R_input”. Those files were then further processed in R (multiple versions) to generate DNA sequence percentages using the code contained in folder “3_R_processing”. Note: Demultiplexing requires QIIME mapping files for each sample plate which are stored in the figshare folder^35^“QIIME_Mapping_Files” for sequence data stored the NCBI SRA database^36^. A complete list of the bioinformatic functions in MacQIIME is available online http://qiime.org/scripts/.

## Online-only Tables

**Online-only Table 1:** The BioProject and associated BioSamples containing the raw sequence data from all seal scat samples archived in the NCBI Sequence Read Archive (SRA).

Accession	Data Archive	BioSample.name	Title	SRA.filename
PRJNA625263	BioProject		Harbour seal DNA metabarcoding diet data of the Salish Sea	
SAMN14596071	BioSample	BC_2012-13_R1_P1_CHCE	AMPLICONS of Phoca vitulina feces: 16S	BC_2012-13_R1_P1_CHCE.fastq
SAMN14596130	BioSample	BC_2012-13_R1_P1_COI	AMPLICONS of Phoca vitulina feces: COI	BC_2012-13_R1_P1_COI.fastq
SAMN14596072	BioSample	BC_2012-13_R1_P2_CHCE	AMPLICONS of Phoca vitulina feces: 16S	BC_2012-13_R1_P2_CHCE.fastq
SAMN14596131	BioSample	BC_2012-13_R1_P2_COI	AMPLICONS of Phoca vitulina feces: COI	BC_2012-13_R1_P2_COI.fastq
SAMN14596073	BioSample	BC_2012-13_R2_P1_CHCE	AMPLICONS of Phoca vitulina feces: 16S	BC_2012-13_R2_P1_CHCE.fastq
SAMN14596132	BioSample	BC_2012-13_R2_P1_COI	AMPLICONS of Phoca vitulina feces: COI	BC_2012-13_R2_P1_COI.fastq
SAMN14596074	BioSample	BC_2012-13_R2_P2_CHCE	AMPLICONS of Phoca vitulina feces: 16S	BC_2012-13_R2_P2_CHCE.fastq
SAMN14596133	BioSample	BC_2012-13_R2_P2_COI	AMPLICONS of Phoca vitulina feces: COI	BC_2012-13_R2_P2_COI.fastq
SAMN14596075	BioSample	BC_2012-13_R2_P3_CHCE	AMPLICONS of Phoca vitulina feces: 16S	BC_2012-13_R2_P3_CHCE.fastq
SAMN14596134	BioSample	BC_2012-13_R2_P3_COI	AMPLICONS of Phoca vitulina feces: COI	BC_2012-13_R2_P3_COI.fastq
SAMN14596076	BioSample	BC_2012-13_R3_P1_CHCE	AMPLICONS of Phoca vitulina feces: 16S	BC_2012-13_R3_P1_CHCE.fastq
SAMN14596135	BioSample	BC_2012-13_R3_P1_COI	AMPLICONS of Phoca vitulina feces: COI	BC_2012-13_R3_P1_COI.fastq
SAMN14596077	BioSample	BC_2012-13_R3_P2_CHCE	AMPLICONS of Phoca vitulina feces: 16S	BC_2012-13_R3_P2_CHCE.fastq
SAMN14596136	BioSample	BC_2012-13_R3_P2_COI	AMPLICONS of Phoca vitulina feces: COI	BC_2012-13_R3_P2_COI.fastq
SAMN14596078	BioSample	BC_2012-13_R3_P3_CHCE	AMPLICONS of Phoca vitulina feces: 16S	BC_2012-13_R3_P3_CHCE.fastq
SAMN14596137	BioSample	BC_2012-13_R3_P3_COI	AMPLICONS of Phoca vitulina feces: COI	BC_2012-13_R3_P3_COI.fastq
SAMN14596079	BioSample	BC_2012-13_R4_P1_CHCE	AMPLICONS of Phoca vitulina feces: 16S	BC_2012-13_R4_P1_CHCE.fastq
SAMN14596138	BioSample	BC_2012-13_R4_P1_COI	AMPLICONS of Phoca vitulina feces: COI	BC_2012-13_R4_P1_COI.fastq
SAMN14596080	BioSample	BC_2012-13_R4_P2_CHCE	AMPLICONS of Phoca vitulina feces: 16S	BC_2012-13_R4_P2_CHCE.fastq
SAMN14596139	BioSample	BC_2012-13_R4_P2_COI	AMPLICONS of Phoca vitulina feces: COI	BC_2012-13_R4_P2_COI.fastq
SAMN14596081	BioSample	BC_2012-13_R5_P1_CHCE	AMPLICONS of Phoca vitulina feces: 16S	BC_2012-13_R5_P1_CHCE.fastq
SAMN14596082	BioSample	BC_2012-13_R5_P2_CHCE	AMPLICONS of Phoca vitulina feces: 16S	BC_2012-13_R5_P2_CHCE.fastq
SAMN14596083	BioSample	BC_2012-13_R5_P3_CHCE	AMPLICONS of Phoca vitulina feces: 16S	BC_2012-13_R5_P3_CHCE.fastq
SAMN14596084	BioSample	BC_2012-13_R5_P4_CHCE	AMPLICONS of Phoca vitulina feces: 16S	BC_2012-13_R5_P4_CHCE.fastq
SAMN14596085	BioSample	BC_2012-13_R6_P1_CHCE	AMPLICONS of Phoca vitulina feces: 16S	BC_2012-13_R6_P1_CHCE.fastq
SAMN14596086	BioSample	BC_2012-13_R6_P2_CHCE	AMPLICONS of Phoca vitulina feces: 16S	BC_2012-13_R6_P2_CHCE.fastq
SAMN14596087	BioSample	BC_2014_R1_P1_CHCE	AMPLICONS of Phoca vitulina feces: 16S	BC_2014_R1_P1_CHCE.fastq
SAMN14596140	BioSample	BC_2014_R1_P1_COI	AMPLICONS of Phoca vitulina feces: COI	BC_2014_R1_P1_COI.fastq
SAMN14596088	BioSample	BC_2014_R1_P2_CHCE	AMPLICONS of Phoca vitulina feces: 16S	BC_2014_R1_P2_CHCE.fastq
SAMN14596141	BioSample	BC_2014_R1_P2_COI	AMPLICONS of Phoca vitulina feces: COI	BC_2014_R1_P2_COI.fastq
SAMN14596089	BioSample	BC_2014_R1_P3_CHCE	AMPLICONS of Phoca vitulina feces: 16S	BC_2014_R1_P3_CHCE.fastq
SAMN14596142	BioSample	BC_2014_R1_P3_COI	AMPLICONS of Phoca vitulina feces: COI	BC_2014_R1_P3_COI.fastq
SAMN14596090	BioSample	BC_2015-16_R1_P1_CHCE	AMPLICONS of Phoca vitulina feces: 16S	BC_2015-16_R1_P1_CHCE.fastq
SAMN14596143	BioSample	BC_2015-16_R1_P1_COI	AMPLICONS of Phoca vitulina feces: COI	BC_2015-16_R1_P1_COI.fastq
SAMN14596091	BioSample	BC_2015-16_R1_P2_CHCE	AMPLICONS of Phoca vitulina feces: 16S	BC_2015-16_R1_P2_CHCE.fastq
SAMN14596144	BioSample	BC_2015-16_R1_P2_COI	AMPLICONS of Phoca vitulina feces: COI	BC_2015-16_R1_P2_COI.fastq
SAMN14596092	BioSample	BC_2015-16_R1_P3_CHCE	AMPLICONS of Phoca vitulina feces: 16S	BC_2015-16_R1_P3_CHCE.fastq
SAMN14596145	BioSample	BC_2015-16_R1_P3_COI	AMPLICONS of Phoca vitulina feces: COI	BC_2015-16_R1_P3_COI.fastq
SAMN14596093	BioSample	BC_2015-16_R3_P1_CHCE	AMPLICONS of Phoca vitulina feces: 16S	BC_2015-16_R3_P1_CHCE.fastq
SAMN14596146	BioSample	BC_2015-16_R3_P1_COI	AMPLICONS of Phoca vitulina feces: COI	BC_2015-16_R3_P1_COI.fastq
SAMN14596094	BioSample	BC_2015-16_R3_P2_CHCE	AMPLICONS of Phoca vitulina feces: 16S	BC_2015-16_R3_P2_CHCE.fastq
SAMN14596147	BioSample	BC_2015-16_R3_P2_COI	AMPLICONS of Phoca vitulina feces: COI	BC_2015-16_R3_P2_COI.fastq
SAMN14596095	BioSample	BC_2015-16_R3_P3_CHCE	AMPLICONS of Phoca vitulina feces: 16S	BC_2015-16_R3_P3_CHCE.fastq
SAMN14596148	BioSample	BC_2015-16_R3_P3_COI	AMPLICONS of Phoca vitulina feces: COI	BC_2015-16_R3_P3_COI.fastq
SAMN14596096	BioSample	BC_2015-16_R4_P1_CHCE	AMPLICONS of Phoca vitulina feces: 16S	BC_2015-16_R4_P1_CHCE.fastq
SAMN14596149	BioSample	BC_2015-16_R4_P1_COI	AMPLICONS of Phoca vitulina feces: COI	BC_2015-16_R4_P1_COI.fastq
SAMN14596097	BioSample	BC_2015-16_R4_P2_CHCE	AMPLICONS of Phoca vitulina feces: 16S	BC_2015-16_R4_P2_CHCE.fastq
SAMN14596150	BioSample	BC_2015-16_R4_P2_COI	AMPLICONS of Phoca vitulina feces: COI	BC_2015-16_R4_P2_COI.fastq
SAMN14596098	BioSample	BC_2015-16_R4_P3_CHCE	AMPLICONS of Phoca vitulina feces: 16S	BC_2015-16_R4_P3_CHCE.fastq
SAMN14596151	BioSample	BC_2015-16_R4_P3_COI	AMPLICONS of Phoca vitulina feces: COI	BC_2015-16_R4_P3_COI.fastq
SAMN14596099	BioSample	BC_2015-16_R5_P1_CHCE	AMPLICONS of Phoca vitulina feces: 16S	BC_2015-16_R5_P1_CHCE.fastq
SAMN14596152	BioSample	BC_2015-16_R5_P1_COI	AMPLICONS of Phoca vitulina feces: COI	BC_2015-16_R5_P1_COI.fastq
SAMN14596100	BioSample	BC_2015-16_R5_P2_CHCE	AMPLICONS of Phoca vitulina feces: 16S	BC_2015-16_R5_P2_CHCE.fastq
SAMN14596153	BioSample	BC_2015-16_R5_P2_COI	AMPLICONS of Phoca vitulina feces: COI	BC_2015-16_R5_P2_COI.fastq
SAMN14596101	BioSample	BC_2015-16_R5_P3_CHCE	AMPLICONS of Phoca vitulina feces: 16S	BC_2015-16_R5_P3_CHCE.fastq
SAMN14596154	BioSample	BC_2015-16_R5_P3_COI	AMPLICONS of Phoca vitulina feces: COI	BC_2015-16_R5_P3_COI.fastq
**Accession**	**Data Archive**	**BioSample.name**	**Title**	**SRA.filename**
SAMN14596102	BioSample	WA_NPS_2016_R1_P1_CHCE	AMPLICONS of Phoca vitulina feces: 16S	WA_NPS_2016_R1_P1_CHCE.fastq
SAMN14596155	BioSample	WA_NPS_2016_R1_P1_COI	AMPLICONS of Phoca vitulina feces: COI	WA_NPS_2016_R1_P1_COI.fastq
SAMN14596103	BioSample	WA_NPS_2016_R1_P2_CHCE	AMPLICONS of Phoca vitulina feces: 16S	WA_NPS_2016_R1_P2_CHCE.fastq
SAMN14596156	BioSample	WA_NPS_2016_R1_P2_COI	AMPLICONS of Phoca vitulina feces: COI	WA_NPS_2016_R1_P2_COI.fastq
SAMN14596104	BioSample	WA_NPS_2016_R1_P3_CHCE	AMPLICONS of Phoca vitulina feces: 16S	WA_NPS_2016_R1_P3_CHCE.fastq
SAMN14596157	BioSample	WA_NPS_2016_R1_P3_COI	AMPLICONS of Phoca vitulina feces: COI	WA_NPS_2016_R1_P3_COI.fastq
SAMN14596105	BioSample	WA_NPS_2016_R2_P1_CHCE	AMPLICONS of Phoca vitulina feces: 16S	WA_NPS_2016_R2_P1_CHCE.fastq
SAMN14596158	BioSample	WA_NPS_2016_R2_P1_COI	AMPLICONS of Phoca vitulina feces: COI	WA_NPS_2016_R2_P1_COI.fastq
SAMN14596106	BioSample	WA_NPS_2016_R2_P2_CHCE	AMPLICONS of Phoca vitulina feces: 16S	WA_NPS_2016_R2_P2_CHCE.fastq
SAMN14596159	BioSample	WA_NPS_2016_R2_P2_COI	AMPLICONS of Phoca vitulina feces: COI	WA_NPS_2016_R2_P2_COI.fastq
SAMN14596107	BioSample	WA_NPS_2016_R2_P3_CHCE	AMPLICONS of Phoca vitulina feces: 16S	WA_NPS_2016_R2_P3_CHCE.fastq
SAMN14596160	BioSample	WA_NPS_2016_R2_P3_COI	AMPLICONS of Phoca vitulina feces: COI	WA_NPS_2016_R2_P3_COI.fastq
SAMN14596108	BioSample	WA_SPS_2016_R1_P1_CHCE	AMPLICONS of Phoca vitulina feces: 16S	WA_SPS_2016_R1_P1_CHCE.fastq
SAMN14596161	BioSample	WA_SPS_2016_R1_P1_COI	AMPLICONS of Phoca vitulina feces: COI	WA_SPS_2016_R1_P1_COI.fastq
SAMN14596109	BioSample	WA_SPS_2016_R1_P2_CHCE	AMPLICONS of Phoca vitulina feces: 16S	WA_SPS_2016_R1_P2_CHCE.fastq
SAMN14596162	BioSample	WA_SPS_2016_R1_P2_COI	AMPLICONS of Phoca vitulina feces: COI	WA_SPS_2016_R1_P2_COI.fastq
SAMN14596110	BioSample	WA_SPS_2016_R2_P1_CHCE	AMPLICONS of Phoca vitulina feces: 16S	WA_SPS_2016_R2_P1_CHCE.fastq
SAMN14596163	BioSample	WA_SPS_2016_R2_P1_COI	AMPLICONS of Phoca vitulina feces: COI	WA_SPS_2016_R2_P1_COI.fastq
SAMN14596111	BioSample	WA_SPS_2016_R2_P2_CHCE	AMPLICONS of Phoca vitulina feces: 16S	WA_SPS_2016_R2_P2_CHCE.fastq
SAMN14596164	BioSample	WA_SPS_2016_R2_P2_COI	AMPLICONS of Phoca vitulina feces: COI	WA_SPS_2016_R2_P2_COI.fastq
SAMN14596112	BioSample	WA_SPS_2016_R3_P1_CHCE	AMPLICONS of Phoca vitulina feces: 16S	WA_SPS_2016_R3_P1_CHCE.fastq
SAMN14596165	BioSample	WA_SPS_2016_R3_P1_COI	AMPLICONS of Phoca vitulina feces: COI	WA_SPS_2016_R3_P1_COI.fastq
SAMN14596113	BioSample	WA_SPS_2016_R3_P2_CHCE	AMPLICONS of Phoca vitulina feces: 16S	WA_SPS_2016_R3_P2_CHCE.fastq
SAMN14596166	BioSample	WA_SPS_2016_R3_P2_COI	AMPLICONS of Phoca vitulina feces: COI	WA_SPS_2016_R3_P2_COI.fastq
SAMN14596114	BioSample	WA_SPS_2016_R4_P1_CHCE	AMPLICONS of Phoca vitulina feces: 16S	WA_SPS_2016_R4_P1_CHCE.fastq
SAMN14596167	BioSample	WA_SPS_2016_R4_P1_COI	AMPLICONS of Phoca vitulina feces: COI	WA_SPS_2016_R4_P1_COI.fastq
SAMN14596115	BioSample	WA_SPS_2016_R4_P2_CHCE	AMPLICONS of Phoca vitulina feces: 16S	WA_SPS_2016_R4_P2_CHCE.fastq
SAMN14596168	BioSample	WA_SPS_2016_R4_P2_COI	AMPLICONS of Phoca vitulina feces: COI	WA_SPS_2016_R4_P2_COI.fastq
SAMN14596116	BioSample	WA_SPS_2016_R5_P1_CHCE	AMPLICONS of Phoca vitulina feces: 16S	WA_SPS_2016_R5_P1_CHCE.fastq
SAMN14596169	BioSample	WA_SPS_2016_R5_P1_COI	AMPLICONS of Phoca vitulina feces: COI	WA_SPS_2016_R5_P1_COI.fastq
SAMN14596117	BioSample	WA_SPS_2016_R5_P2_CHCE	AMPLICONS of Phoca vitulina feces: 16S	WA_SPS_2016_R5_P2_CHCE.fastq
SAMN14596170	BioSample	WA_SPS_2016_R5_P2_COI	AMPLICONS of Phoca vitulina feces: COI	WA_SPS_2016_R5_P2_COI.fastq
SAMN14596118	BioSample	WA_SPS_2017-18_R1_P1_CHCE	AMPLICONS of Phoca vitulina feces: 16S	WA_SPS_2017-18_R1_P1_CHCE.fastq
SAMN14596171	BioSample	WA_SPS_2017-18_R1_P1_COI	AMPLICONS of Phoca vitulina feces: COI	WA_SPS_2017-18_R1_P1_COI.fastq
SAMN14596119	BioSample	WA_SPS_2017-18_R1_P2_CHCE	AMPLICONS of Phoca vitulina feces: 16S	WA_SPS_2017-18_R1_P2_CHCE.fastq
SAMN14596172	BioSample	WA_SPS_2017-18_R1_P2_COI	AMPLICONS of Phoca vitulina feces: COI	WA_SPS_2017-18_R1_P2_COI.fastq
SAMN14596120	BioSample	WA_SPS_2017-18_R2_P1_CHCE	AMPLICONS of Phoca vitulina feces: 16S	WA_SPS_2017-18_R2_P1_CHCE.fastq
SAMN14596173	BioSample	WA_SPS_2017-18_R2_P1_COI	AMPLICONS of Phoca vitulina feces: COI	WA_SPS_2017-18_R2_P1_COI.fastq
SAMN14596121	BioSample	WA_SPS_2017-18_R2_P2_CHCE	AMPLICONS of Phoca vitulina feces: 16S	WA_SPS_2017-18_R2_P2_CHCE.fastq
SAMN14596174	BioSample	WA_SPS_2017-18_R2_P2_COI	AMPLICONS of Phoca vitulina feces: COI	WA_SPS_2017-18_R2_P2_COI.fastq
SAMN14596122	BioSample	WA_SPS_2017-18_R3_P1_CHCE	AMPLICONS of Phoca vitulina feces: 16S	WA_SPS_2017-18_R3_P1_CHCE.fastq
SAMN14596175	BioSample	WA_SPS_2017-18_R3_P1_COI	AMPLICONS of Phoca vitulina feces: COI	WA_SPS_2017-18_R3_P1_COI.fastq
SAMN14596123	BioSample	WA_SPS_2017-18_R3_P2_CHCE	AMPLICONS of Phoca vitulina feces: 16S	WA_SPS_2017-18_R3_P2_CHCE.fastq
SAMN14596176	BioSample	WA_SPS_2017-18_R3_P2_COI	AMPLICONS of Phoca vitulina feces: COI	WA_SPS_2017-18_R3_P2_COI.fastq
SAMN14596124	BioSample	WA_SPS_2017-18_R4_P1_CHCE	AMPLICONS of Phoca vitulina feces: 16S	WA_SPS_2017-18_R4_P1_CHCE.fastq
SAMN14596177	BioSample	WA_SPS_2017-18_R4_P1_COI	AMPLICONS of Phoca vitulina feces: COI	WA_SPS_2017-18_R4_P1_COI.fastq
SAMN14596125	BioSample	WA_SPS_2017-18_R4_P2_CHCE	AMPLICONS of Phoca vitulina feces: 16S	WA_SPS_2017-18_R4_P2_CHCE.fastq
SAMN14596178	BioSample	WA_SPS_2017-18_R4_P2_COI	AMPLICONS of Phoca vitulina feces: COI	WA_SPS_2017-18_R4_P2_COI.fastq
SAMN14596126	BioSample	WA_SPS_2017-18_R5_P1_CHCE	AMPLICONS of Phoca vitulina feces: 16S	WA_SPS_2017-18_R5_P1_CHCE.fastq
SAMN14596179	BioSample	WA_SPS_2017-18_R5_P1_COI	AMPLICONS of Phoca vitulina feces: COI	WA_SPS_2017-18_R5_P1_COI.fastq
SAMN14596127	BioSample	WA_SPS_2017-18_R5_P2_CHCE	AMPLICONS of Phoca vitulina feces: 16S	WA_SPS_2017-18_R5_P2_CHCE.fastq
SAMN14596180	BioSample	WA_SPS_2017-18_R5_P2_COI	AMPLICONS of Phoca vitulina feces: COI	WA_SPS_2017-18_R5_P2_COI.fastq
SAMN14596128	BioSample	WA_SPS_2017-18_R6_P1_CHCE	AMPLICONS of Phoca vitulina feces: 16S	WA_SPS_2017-18_R6_P1_CHCE.fastq
SAMN14596181	BioSample	WA_SPS_2017-18_R6_P1_COI	AMPLICONS of Phoca vitulina feces: COI	WA_SPS_2017-18_R6_P1_COI.fastq
SAMN14596129	BioSample	WA_SPS_2017-18_R6_P2_CHCE	AMPLICONS of Phoca vitulina feces: 16S	WA_SPS_2017-18_R6_P2_CHCE.fastq
SAMN14596182	BioSample	WA_SPS_2017-18_R6_P2_COI	AMPLICONS of Phoca vitulina feces: COI	WA_SPS_2017-18_R6_P2_COI.fastq

Each.fastq file contains data from one sequencing plate of 96 scat samples. QIIME mapping files for demultiplexing are available in the fig**share** archive.

**Online-only Table 2:** Table 5.1 from Thomas (2015).

Site	Fraser River								Comox								Cowichan Bay								Belle Chain			
year	2012				2013				2012				2013				2012				2013				2012		2013	
season	Spring		Fall		Spring		Fall		Spring		Fall		Spring		Fall		Spring		Fall		Spring		Fall		Fall		Fall	
method	DNA	HP	DNA	HP	DNA	HP	DNA	HP	DNA	HP	DNA	HP	DNA	HP	DNA	HP	DNA	HP	DNA	HP	DNA	HP	DNA	HP	DNA	HP	DNA	HP
sample size	70	67	83	83	88	101	70	70	85	79	111	119	98	106	73	73	56	53	83	85	76	86	91	95	85	87	77	81
Herring spp.	—	12.0	—	1.6	—	10.1	—	0.5	—	10.3	—	1.5	—	5.6	—	5.4	—	8.3	—	4.4	—	6.7	—	4.5	—	10.4	—	5.1
Pacific herring	16.1	7.6	2.2	0.7	14.0	10.4	3.6	0.7	36.1	26.0	23.8	20.5	36.7	39.3	22.9	19.0	48.1	30.1	24.7	16.8	42.4	47.4	26.2	23.1	44.1	29.8	31.6	23.6
American shad	4.2	—	—	—	0.4	—	—	—	0.4	—	—	—	—	—	—	—	—	—	—	—	—	—	—	—	0.8	—	—	—
Pacific sardine	—	1.6	—	—	—	—	—	—	—	—	—	—	—	—	—	—	—	0.9	—	—	—	—	—	—	—	0.6	—	—
**Herring total**	**20.3**	**21.2**	**2.2**	**2.3**	**14.3**	**20.6**	**3.6**	**1.2**	**36.6**	**36.3**	**23.8**	**22.0**	**36.7**	**44.9**	**22.9**	**24.4**	**48.1**	**39.4**	**24.7**	**21.2**	**42.4**	**54.1**	**26.2**	**27.6**	**44.9**	**40.8**	**31.6**	**28.7**
Salmon spp. adult	—	32.2	—	79.9	—	4.7	—	83.6	—	2.3	—	28.5	—	0.3	—	29.8	—	—	—	20.1	—	1.6	—	14.7	—	6.0	—	40.9
Chinook salmon ad.	4.6	—	22.7	—	6.5	—	4.0	—	2.4	—	2.3	—	1.0	—	2.5	—	—	—	9.0	—	0.8	—	3.4	—	4.1	—	2.2	—
chum salmon ad.	0.1	—	38.4	—	0.3	—	20.6	—	—	—	24.1	—	—	—	4.4	—	—	—	11.7	—	—	—	9.4	—	—	—	7.7	—
coho salmon ad.	0.4	—	2.0	—	—	—	2.5	—	—	—	0.8	—	—	—	1.4	—	—	—	0.5	—	0.3	—	2.5	—	0.4	—	0.5	—
pink salmon ad.	0.6	—	1.4	—	0.1	—	45.5	—	0.3	—	3.4	—	—	—	26.8	—	—	—	2.1	—	—	—	5.9	—	0.5	—	35.8	—
sockeye salmon ad.	35.7	—	23.0	—	1.6	—	18.6	—	0.6	—	0.9	—	0.3	—	2.7	—	—	—	3.2	—	—	—	2.3	—	2.8	—	4.3	—
steelhead ad.	1.6	—	—	—	—	—	—	—	—	—	—	—	—	—	—	—	—	—	0.2	—	—	—	—	—	—	—	—	—
Salmon spp. juvenile	—	1.9	—	0.9	—	1.5	—	0.5	—	10.1	—	2.8	—	5.2	—	4.2	—	4.8	—	3.4	—	1.8	—	3.1	—	7.1	—	3.0
Chinook salmon juv.	2.3	—	0.5	—	2.8	—	—	—	4.5	—	0.1	—	0.8	—	0.3	—	6.2	—	2.3	—	2.3	—	1.8	—	7.5	—	—	—
chum salmon juv.	0.2	—	0.2	—	0.4	—	1.3	—	0.9	—	0.6	—	2.2	—	0.1	—	—	—	0.5	—	0.5	—	1.0	—	0.4	—	1.3	—
coho salmon juv.	0.6	—	—	—	0.9	—	—	—	5.0	—	0.1	—	4.3	—	2.6	—	2.8	—	0.6	—	3.2	—	0.2	—	0.6	—	0.8	—
pink salmon juv.	0.3	—	0.1	—	2.2	—	—	—	1.8	—	1.0	—	0.5	—	2.2	—	0.8	—	1.2	—	2.6	—	0.9	—	0.5	—	0.3	—
sockeye salmon juv.	0.1	—	—	—	2.9	—	—	—	1.3	—	0.3	—	4.4	—	1.4	—	2.8	—	1.3	—	2.7	—	0.9	—	5.4	—	—	—
steelhead juv.	0.5	—	—	—	—	—	—	—	1.3	—	—	—	—	—	—	—	—	—	0.1	—	—	—	—	—	0.1	—	—	—
**Salmon total**	**47.0**	**34.1**	**88.4**	**80.8**	**17.6**	**6.2**	**92.5**	**84.0**	**18.1**	**12.5**	**33.6**	**31.3**	**13.4**	**5.5**	**44.4**	**34.0**	**12.7**	**4.8**	**32.9**	**23.4**	**12.4**	**3.3**	**28.3**	**17.8**	**22.3**	**13.1**	**52.9**	**43.9**
Codfish spp.	—	3.4	—	—	—	5.2	—	2.1	—	4.0	—	1.3	—	1.5	—	1.8	—	4.1	—	4.9	—	5.5	—	4.2	—	2.8	—	—
walleye pollock	5.8	5.5	1.3	0.2	8.2	10.8	0.5	2.1	8.1	3.0	2.6	3.9	1.6	0.7	0.5	2.2	11.0	7.5	13.2	12.0	9.4	8.1	7.3	3.5	21.6	23.1	9.9	8.6
Pacific hake	1.5	1.4	0.6	1.0	6.3	1.4	0.1	—	10.6	9.0	6.7	8.5	25.8	21.2	7.7	6.3	6.9	3.8	12.7	9.1	17.5	7.8	19.6	12.8	1.1	1.6	0.6	0.7
Pacific cod	1.2	—	—	—	0.5	—	—	—	1.0	—	—	—	—	—	0.3	—	0.3	—	—	—	—	—	—	—	1.2	—	—	—
Pacific tomcod	—	—	—	—	—	—	—	—	1.0	2.4	—	0.6	—	—	1.0	—	—	—	—	0.2	—	—	—	—	—	—	—	—
**Gadoids total**	**8.5**	**10.2**	**1.9**	**1.2**	**15.0**	**17.4**	**0.6**	**4.3**	**20.7**	**18.4**	**9.4**	**14.3**	**27.4**	**23.4**	**9.4**	**10.3**	**18.2**	**15.3**	**25.9**	**26.2**	**26.9**	**21.5**	**26.8**	**20.6**	**23.8**	**27.6**	**10.5**	**9.4**
threespine stickleback	3.8	4.7	—	—	21.6	19.1	—	—	0.8	1.9	0.2	—	2.5	0.8	0.1	—	3.8	4.5	—	0.3	5.9	3.2	0.8	0.6	—	0.4	—	—
shiner surfperch	—	—	0.2	0.3	0.4	0.5	0.3	0.5	0.2	1.1	3.8	4.7	3.2	2.9	8.7	8.8	0.5	3.5	3.9	9.1	4.9	3.9	10.7	9.9	—	0.4	0.7	—
Pacific sand lance	0.1	—	—	1.0	0.4	1.4	1.3	1.7	2.4	5.6	2.6	2.8	0.1	2.6	—	—	—	1.3	—	—	—	1.0	—	—	0.2	1.0	—	0.2
snake prickleback	0.2	—	—	0.6	—	0.3	—	—	5.2	5.5	7.8	6.4	1.9	1.7	3.7	3.4	1.7	2.0	—	1.0	—	0.7	—	0.4	—	—	—	—
Smelt spp.	—	4.5	—	0.3	—	9.4	—	—	—	1.3	—	—	—	—	—	—	—	—	—	—	—	—	—	—	—	0.2	—	0.4
Northern smoothtongue	4.9	—	—	—	3.9	1.1	—	—	0.3	—	—	—	0.1	—	—	—	0.6	—	—	—	0.2	—	—	—	—	—	0.6	—
eulachon	0.1	—	—	—	16.8	—	—	—	—	—	0.3	—	0.6	—	—	—	—	—	—	—	0.7	—	—	—	—	—	0.4	—
capelin	—	—	—	—	1.0	5.6	—	—	—	—	—	—	—	—	—	—	—	—	—	—	—	—	—	0.2	—	—	—	—
Online-only Table 2 (continued).																												
**Site**	**Fraser River**								**Comox**								**Cowichan Bay**								**Belle Chain**			
**year**	**2012**				**2013**				**2012**				**2013**				**2012**				**2013**				**2012**		**2013**	
**season**	**Spring**		**Fall**		**Spring**		**Fall**		**Spring**		**Fall**		**Spring**		**Fall**		**Spring**		**Fall**		**Spring**		**Fall**		**Fall**		**Fall**	
**method**	**DNA**	**HP**	**DNA**	**HP**	**DNA**	**HP**	**DNA**	**HP**	**DNA**	**HP**	**DNA**	**HP**	**DNA**	**HP**	**DNA**	**HP**	**DNA**	**HP**	**DNA**	**HP**	**DNA**	**HP**	**DNA**	**HP**	**DNA**	**HP**	**DNA**	**HP**
**sample size**	**70**	**67**	**83**	**83**	**88**	**101**	**70**	**70**	**85**	**79**	**111**	**119**	**98**	**106**	**73**	**73**	**56**	**53**	**83**	**85**	**76**	**86**	**91**	**95**	**85**	**87**	**77**	**81**
Sculpin spp.	—	0.7	—	—	—	—	—	—	—	—	—	3.0	—	—	—	0.5	—	1.8	—	2.0	—	—	—	0.5	—	—	—	—
Pacific staghorn sculpin	—	0.5	1.7	1.0	0.4	0.2	1.4	1.2	0.6	0.4	5.9	4.7	1.3	1.4	3.3	6.2	3.1	1.4	3.4	3.6	0.2	0.2	2.9	3.9	0.2	—	0.2	—
buffalo sculpin	—	—	—	—	—	—	—	—	—	—	—	—	—	—	1.4	—	—	—	2.5	—	—	—	0.4	—	0.2	—	—	—
tadpole sculpin	—	—	—	—	—	—	—	—	—	—	—	—	—	—	—	—	1.9	—	—	—	—	—	—	—	—	—	—	—
blackbelly eelpout	—	0.4	—	—	—	0.3	—	—	1.2	0.4	1.8	1.1	—	0.2	—	0.5	0.1	—	—	0.8	—	—	—	—	—	0.2	—	—
Rockfish spp.	—	—	—	—	—	—	—	—	—	—	—	—	—	—	—	0.8	—	—	—	0.6	—	1.2	—	1.1	—	—	—	—
China rockfish	—	—	—	—	—	—	—	—	—	—	—	—	—	—	—	—	—	—	0.4	—	2.3	—	0.1	—	—	—	—	—
lingcod	0.2	—	—	—	—	—	—	—	2.5	—	4.5	—	—	—	1.9	—	0.4	—	1.5	—	0.3	—	0.1	—	0.4	—	—	—
whitespotted greenling	—	—	—	—	—	—	—	—	0.9	—	2.1	—	—	—	—	—	—	—	1.2	—	—	—	—	—	—	—	—	—
plainfin midshipmen	—	—	—	—	—	0.3	—	—	0.2	0.3	—	0.3	2.0	1.9	0.1	—	2.3	1.9	—	0.4	1.0	—	1.1	0.5	—	—	—	—
cabezon	—	—	—	—	—	—	—	—	—	—	—	—	1.5	—	—	—	—	—	—	—	—	—	—	—	—	—	—	—
Righteye flounder spp.	—	2.9	—	1.4	—	2.9	—	0.5	—	1.3	—	1.1	—	6.6	—	2.3	—	0.6	—	—	—	3.2	—	1.6	—	0.4	—	—
starry flounder	—	—	2.4	1.9	—	0.7	—	2.1	2.9	1.7	0.9	2.7	2.1	2.1	2.2	2.7	1.8	0.6	0.4	0.4	—	—	0.1	—	—	—	—	—
English sole	0.9	0.4	1.7	0.3	0.2	1.5	—	—	1.0	1.7	1.7	1.0	2.2	—	0.3	0.2	1.7	1.9	0.2	—	—	—	—	—	0.1	—	—	—
Dover sole	0.6	—	—	—	0.9	—	—	—	—	—	—	—	—	—	—	—	—	—	—	—	1.4	—	0.9	—	—	—	—	—
arrowtooth flounder	—	—	—	—	—	—	—	—	0.3	—	0.1	—	2.0	—	1.3	—	—	—	—	—	—	—	—	—	—	—	—	—
Pacific halibut	1.9	—	0.1	—	—	—	0.2	—	—	—	—	—	—	—	—	—	—	—	—	—	0.2	—	—	—	—	—	—	—
Unidentifiable fish spp.	—	12.2	—	2.4	—	2.5	—	2.9	—	3.2	—	0.8	—	2.4	—	—	—	3.8	—	2.9	—	0.7	—	2.1	—	3.4	—	2.5
Unknown fish spp.	—	—	—	2.4	—	—	—	—	—	1.3	—	2.4	—	0.2	—	1.4	—	—	—	0.6	—	0.4	—	—	—	—	—	1.2
river lamprey	1.6	2.8	0.1	1.4	0.3	3.4	—	1.7	0.7	—	—	—	—	—	—	—	—	1.1	1.3	1.0	—	1.0	0.1	3.3	4.0	2.8	1.8	2.4
spotted ratfish	4.1	—	0.6	—	2.5	—	—	—	0.1	—	0.9	—	—	—	—	—	1.7	—	0.4	—	0.2	—	—	—	2.7	—	—	—
Skate spp.	—	2.6	—	—	—	2.8	—	—	—	—	—	—	—	0.3	—	0.2	—	—	—	—	—	—	—	—	—	0.6	—	—
big skate	3.3	—	—	—	3.2	—	—	—	0.4	—	—	—	1.1	—	0.2	—	—	—	—	—	—	—	—	—	—	—	—	—
magister armhook squid	0.5	1.7	—	1.3	0.1	1.0	—	—	0.3	0.6	—	0.7	—	—	—	0.8	0.2	5.8	0.1	—	—	0.2	—	1.1	0.2	5.9	—	5.7
California market squid	0.2	—	—	—	—	0.2	—	—	2.4	2.7	—	—	—	1.5	—	—	0.6	4.1	0.2	2.0	—	1.4	—	2.2	—	—	—	—
clawed armhook squid	—	—	—	—	—	—	—	—	—	—	—	—	—	—	—	—	—	5.6	—	1.9	—	0.6	—	—	—	0.4	—	0.9
giant Pacific octopus	—	—	—	—	—	—	—	—	1.4	—	—	—	—	—	—	—	—	—	—	—	0.4	—	—	—	—	—	0.9	—
Unknown cephalopod spp.	—	0.2	—	0.6	—	1.0	—	—	—	1.9	—	0.3	—	0.3	—	—	—	—	—	2.6	—	0.7	—	3.0	—	0.7	—	3.5
Unknown crustacean spp.	—	—	—	—	—	0.2	—	—	—	—	—	—	—	0.8	—	3.2	—	—	—	—	—	0.9	—	2.2	—	—	—	1.2
<1% species	1.3	1.0	0.5	0.6	1.1	1.4	0.1	—	0.7	1.9	0.2	0.6	1.7	0.5	—	0.2	0.5	0.5	0.8	—	0.4	2.0	1.2	1.5	0.7	2.2	0.1	—
**Other total**	**23.8**	**34.5**	**7.4**	**15.6**	**52.8**	**55.8**	**3.2**	**10.5**	**24.4**	**32.8**	**32.9**	**32.4**	**22.3**	**26.2**	**23.2**	**31.3**	**20.8**	**40.4**	**16.2**	**29.1**	**18.1**	**21.2**	**18.5**	**34.1**	**8.6**	**18.5**	**4.8**	**18.0**
**All total**	**100**	**100**	**100**	**100**	**100**	**100**	**100**	**100**	**100**	**100**	**100**	**100**	**100**	**100**	**100**	**100**	**100**	**100**	**100**	**100**	**100**	**100**	**100**	**100**	**100**	**100**	**100**	**100**

Diets of harbour seals (%) stratified by sampling location (Fraser River, Comox, Cowichan Bay, Belle Chain), year and season (spring: Apr-Jul; fall: Aug- Nov). Percentages are estimates of the relative mass consumed and were calculated using DNA metabarcoding RRA (DNA) and prey hardparts Split Sample Frequency of Occurrence (HP). Sample size indicates the number of scats that had useable DNA or hard parts to estimate average diets.
