# Supplementary material for: Data on the diets of Salish Sea harbour seals from DNA metabarcoding
Source: Sci Data. 2022 Mar 2;9:68. doi: 10.1038/s41597-022-01152-5 (PMC8891336; doi:10.1038/s41597-022-01152-5)
Supplement: Supplementary file 1 — Supplementary Information [file 41597_2022_1152_MOESM1_ESM.pdf]

Validation of diet reconstruction (Predation by Harbour Seals on Salmon Smolts Project)

Prepared for Pacific Science Foundation

[July, 2018]

SMRU Consulting North America

PO Box 764
Friday Harbor, WA 98250
USA

1529 West 6th Ave., Suite 510
Vancouver, BC V6J 1R1
Canada

Validation of diet reconstruction (Predation by Harbour Seals on Salmon Smolts Project)

July 2018

Prepared by SMRU Consulting NA

Authors:

Dominic Tollit, PhD
Senior Research Scientist

Ruth Joy, PhD
Senior Research Scientist

For its part, the Buyer acknowledges that Reports supplied by the Seller as part of the Services may be misleading if not read in their entirety, and can misrepresent the position if presented in selectively edited form. Accordingly, the Buyer undertakes that it will make use of Reports only in unedited form, and will use reasonable endeavours to procure that its client under the Main Contract does likewise. As a minimum, a full copy of our Report must be appended to the broader Report to the client.

Table of Contents

1. Introduction **1**
1.1 Project Description **1**
2. Methods **2**
2.1 Scat prey biomass reconstruction and dietary index comparisons **2**
3. Results **1**
3.1 Dietary reconstruction based on prey hard parts **1**
3.2. Dietary reconstruction based on DNA **3**
3.3. Dietary reconstruction comparisons **3**
3.4. Effects of scat sub sampling protocols on dietary reconstruction **7**
3.5. Statistical Analyses **13**
4. Discussion **13**
Acknowledgements **17**
References **17**

List of Figures

Figure 1 Percent diet contribution by species group comparing diet indices using prey hard parts and prey DNA sequences isolated from scats (median and 2.5%ile and 97.5%ile confidence intervals based on bootstrap resampling with replacement) for DNA-Fixed and Fixed and Variable prey biomass reconstruction (BR) dietary indices without and with numerical correction factors (NCF) applied to hard parts that account for complete prey digestion (Fixed=uses fixed proportions within each scat based on prey biomass reconstruction, Variable=uses prey biomass reconstruction across all scats to determine proportions) 5
Figure 2 Percent diet contribution by species group comparing occurrence indices using prey hard parts and prey DNA isolated from scats for percent modified frequency of occurrence (MFO) and percent split sample frequency of occurrence (SSFO) 6
Figure 3. Percent diet contribution by key species using prey hard parts (HP) and prey DNA isolated from scats comparing occurrence indices (percent modified frequency of occurrence (MFO) and percent split sample frequency of occurrence (SSFO)) and DNA-Fixed and Fixed and Variable prey biomass reconstruction (BR) dietary indices with numerical correction factors applied to hard parts that account for complete prey digestion (Fixed=uses fixed proportions within each scat based on prey biomass reconstruction or on DNA sequencing data, Variable=uses prey biomass reconstruction across all scats to determine proportions) 7
Figure 4 Percent diet contribution by species group for a range of scat sample sizes comparing diet indices using prey hard parts and prey DNA sequences isolated from scats (median and 2.5%ile and 97.5%ile confidence intervals based on bootstrap resampling with replacement) for DNA-Fixed and BR-Fixed with numerical correction factors (NCF) that account for complete prey digestion (Fixed=uses fixed proportions within each scat based on prey biomass reconstruction or on DNA sequencing data) 8

List of Tables

Table 1 Prey size estimation information. FL=fork length, SL=standard length, TL=Total length. DFO = Fisheries and Oceans Canada, Unpubl. Data. Length-Weight allometric regressions follow: Weight = a(prey Length)^{b}.. 1
Table 2. Prey species categories and key prey species 'groups' together with number of prey identifed and Numerical Correction Factors (NCF) by length class (where applicable) applied to prey recovered as hard parts identified in 212 harbor seals scats collected in the Strait of Georgia in 2016. For some species size-specific NCFs are provided. Lower and upper 95% confidence intervals are provided for NCFs, noting values in italics were not derived experimentally............ 2
Table 3. Percent diet contribution by species group using prey hard parts recovered from scats (median i.e. 50%ile and lower 2.5%ile and upper 97.5%ile confidence intervals based on bootstrap resampling with replacement) for prey occurrence dietary indices (MFO=% modified frequency of occurrence, SSFO= % split sample frequency of occurrence). 1
Table 4. Percent diet contribution by species group using prey hard parts recovered from scats (median and 2.5%ile and 97.5%ile confidence intervals based on bootstrap resampling with replacement) for prey biomass reconstruction (BR) dietary indices without numerical correction factors applied to hard parts that account for complete prey digestion (Fixed=uses fixed proportions within each scat based on prey biomass reconstruction, Variable=uses prey biomass reconstruction across all scats to determine proportions) 2
Table 5. Percent diet contribution by species group using prey hard parts recovered from scats (median and 2.5%ile and 97.5%ile confidence intervals based on bootstrap resampling with replacement) for prey biomass reconstruction (BR) dietary indices with numerical correction factors applied to hard parts that account for complete prey digestion (Fixed=uses fixed proportions within each scat based on prey biomass reconstruction, Variable=uses prey biomass reconstruction across all scats to determine proportions) 2
Table 6. Percent diet contribution by species group using DNA isolated from scats (median and 2.5%ile and 97.5%ile confidence intervals based on bootstrap resampling with replacement) for prey occurrence dietary indices (MFO=% modified frequency of occurrence, SSFO= % split sample frequency of occurrence) and DNA sequence-based reconstruction using a Fixed reconstruction methodology (Fixed=uses fixed proportions within each scat based on DNA sequencing data). ... 3
Table 7. Percent diet contribution by common species name using prey hard parts recovered from scats (the 50%ile or median is shaded with 2.5%ile and 97.5%ile confidence intervals based on bootstrap resampling with replacement) for prey occurrence (MFO and SSFO) and biomass reconstruction (BR) dietary indices without and with numerical correction factors (NCF) that account for complete prey digestion (Fixed=uses fixed proportions within each scat based on prey biomass reconstruction, Variable=uses prey biomass reconstruction across all scats to determine proportions). Salmon diet contributions are highlighted in italics and order based on BR-Fixed with C.F. 8
Table 8. Percent diet contribution by common species name using DNA sequence data isolated from scats (the 50%ile or median is shaded with 2.5%ile and 97.5%ile confidence intervals based on bootstrap resampling with replacement) for prey occurrence (MFO and SSFO) and proportions based on prey sequences (DNA-Fixed) (Fixed=uses fixed proportions within each scat based on

prey biomass reconstruction). Salmon diet contributions are highlighted in italics and order based on DNA-Fixed. 10
Table 9. Results of Spearman Rank correlation tests across DNA and hard part indices 13

1. Introduction

Accurate information about what pinnipeds eat is challenging to obtain, yet vital for assessing the impacts of pinnipeds on prey populations and pinniped interactions with fisheries and other predators. Biomass reconstruction using prey hard parts in scats can theoretically provide useful quantitative estimates of diet for pinnipeds (Bowen 2000, Tollit et al. 2007), but certain key concerns have proved hard to solve (Pierce and Boyle 1991, Tollit et al. 2006), particularly the possibility of not detecting (or severely underestimating) important prey contributions. This may occur if soft bodied prey are not represented by hard parts (Olesiuk et al. 1990), if only the fleshy parts of large or spiny prey are consumed (e.g., the bellies of salmon) or if a prey's hard parts are preferentially regurgitated (e.g., cephalopod beaks; see Bigg and Fawcett 1985). Furthermore, prey with robust skeletal elements may be overrepresented compared with prey with fragile skeletons that poorly survive the digestive process (Jobling and Breiby 1986, Murie and Lavigne 1986). In addition, a number of commercially and trophically important prey taxa (notably Salmonidae, Scorpaenidae, and Elasmobranchii) can typically only be identified using hard parts to the family/genera level, rather than the species level. This makes assessing predation levels on specific salmon species highly challenging. Importantly, hard parts can provide estimates of prey size if some account of the level of digestion is made.

Recent advances in molecular technologies have already proven useful in a number of marine mammal dietary studies (e.g., Reed et al. 1997, Jarman et al. 2002, Casper et al. 2007, Tollit et al. 2009), notably by increasing taxon-level detection rates and improving species resolution. Importantly, captive feeding studies have reliably (>95%) detected different prey species fed in varied quantities by extracting prey DNA from scat soft-part matrix (prey flesh remains) and have shown detection of prey is limited to a 48-h period after feeding (Deagle et al. 2005). In contrast, passage times of hard parts are far more variable, especially cephalopod beaks, due to long-term retention in the digestive tract (Bigg and Fawcett 1985, Tollit et al. 2003), complicating accurate diet composition estimation. Overall, molecular approaches have the potential to evaluate and alleviate some of the potential biases and limitations associated with reconstructing diets using hard-part identification (e.g., Casper et al. 2007, Tollit et al. 2009, Tollit et al. 2017). These include the ability to differentiate across salmon species, as well as detect species where hard parts have not been consumed or are highly digestible.

1.1 Project Description

As part of *Study #2 – Validation of diet reconstruction* within the **Predation by Harbour Seals on Salmon Smolts Project**, SMRU Consulting Canada undertook the following 3 tasks;

1) Completed a scat prey biomass reconstruction using approximately 210 scats collected in 2016 during Study 1 of this Project (*Non-estuary seal diet*). This required size estimation using prey species-specific mass-length regression applied to recovered hard-parts and the application of available numerical correction factors (NCFs) to account for complete digestion or loss of prey hard parts.

2) Developed bespoke computer code to estimate diet using both biomass reconstruction and frequency of occurrence methods applied to prey hard parts.
3) Ran Monte Carlo bootstrap simulations on all three diet estimation methods (prey occurrence from prey DNA and prey hard parts identifications, Biomass Reconstruction from prey hard parts, and diet reconstruction quantified using 'relative read abundance' of prey DNA sequence data) using scat data from task 1 to understand the 95% confidence intervals around diet estimates, as well explore biases and limitations of the different methods of reconstructing diets. Work was undertaken in collaboration with Chad Nordstrom, Sheena Majewski, as well as Drs Thomas and Trites.

The goal of the study was to address key questions that have been raised about the methods used to estimate consumption of salmon by seals, specifically comparing biomass reconstruction versus diet quantified using DNA sequencing.

2. Methods

2.1 Scat prey biomass reconstruction and dietary index comparisons

Harbour seal scats were collected in the Strait of Georgia by DFO in 2016. Individual scats were rinsed through mesh strainers and all retained prey hard parts were identified to the lowest possible taxonomic group based on diagnostic morphological criteria developed by Pacific IDentifications Inc. using comprehensive comparative reference skeletons. Scat samples with no prey remains or only the remains of polychaetes or crustaceans were excluded from further dietary analysis. A subsample of 212 scats collected in spring and summer were provided by DFO for prey biomass reconstruction (BR) diet estimation. A total of 96% contained prey (n=204 scats), excluding 5 samples that contained Nereisis sp., which is considered likely secondary prey.

Prey hard part Biomass Reconstruction (BR) requires a number of iterative steps, which fall into two main types; estimating the number and size of prey, followed by diet index modeling. The methods used in this study generally follow those outlined in Tollit et al. (2015; 2017), with supplementary information provided in Table 1. Prey length information was based on size class estimates provided (where possible) from each species by Pacific IDentifications Inc. using reference material. Median prey total lengths were derived within each size class category, noting size class categories were typically 5-12 cm broad and increased as prey size increased. Prey mass was subsequently calculated by applying Prey Length-Prey Mass allometric regressions (Tollit et al. (2015)), with modification to standard length or fork length where necessary using published data or reported values taken from www.fishbase.org. Seven new project-specific regressions based on 2015 and or 2016 DFO fishery data were developed for key prey species, including using survey data specific to the Strait of Georgia for the five most prevalent prey (Pacific hake, Pacific herring, Walleye pollock, Shiner perch and Plainfin midshipman). DFO data was also used to estimate the size of juvenile salmon based on trawl survey data collected in 2016 across the Salish sea. Prey size for low prevalence prey without species-specific size estimates, including skate spp., lamprey spp. and cephalopod spp. were derived from various sources (see Table 1 for details).

Numerical correction factors (NCFs, Tollit et al. 2015 and 2017) were applied to account for total digestion of prey hard parts and where data was available, prey size-specific NCFs were applied (see Tollit et al. 1997; 2007; 2015; 2017; Philips 2005; see Table 2 for details). Due to the lack of studies that derive all structure NCFs for key British Columbia prey species, this study relied on NCFs derived from active sea lion captive feeding studies that collected comparable (i.e., standardized) data across multiple key BC species. Proxy species NCFs were used when no other data existed and based on prey structure robustness (Pers. Comm, Susan Crockford, Pacific IDentifications Inc.). As described in multiple captive pinniped feeding studies (e.g., Bowen 2000), NCFs applied in this study reduced the importance of robust prey species like large gadids and cephalopods, compared to prey species with fragile hard parts like herring, sandlance, anchovy, sardine (forage fish) and salmon. Given the high uncertainty around some NCFs (Table 2), the sensitivity of biomass-based diet estimates was assessed by comparing dietary contributions with and without NCFs applied. For prey hard part versus DNA methodology comparative purposes, dietary contributions of prey species were pooled into 8 key prey groupings (following Trites et al. 2007). These were Gadids, Forage fish, Cephalopods, Flatfish, Hexagrammids (greenlings spp.), Rockfish, Salmon and all 'Other' species (see Table 2 for information on groupings).

Custom R code was developed to provide two reconstructed composite diet model variants (termed 'Variable' and 'Fixed'; *sensu* Laake et al. 2002). Variable diet models allow for variability in foraging success (meal size) between animals as a result of variable biomass contribution across scats (i.e., contribution of prey species to the total depends on the total biomass of prey predicted). Fixed diet models assign the proportion of each prey species within each scat and each scat contributes equally to the total. Proportion of prey biomass ($\hat{\pi}$) was first determined using the variable biomass (BR-Variable) and fixed biomass (BR-Fixed) models (Laake et al. 2002):

BR-V

$$\hat{\pi}_i = \frac{\sum_{k=1}^{s} b_{i,k}}{\sum_{i=1}^{\omega} \sum_{k=1}^{s} b_{i,k}}$$

BR-F

$$\hat{\pi}_i = \frac{\sum_{k=1}^{s} \left(\frac{b_{ik}}{\sum_{i=1}^{w} b_{ik}} \right)}{s}$$

where, b_i = the total biomass of prey type *i*, where there are *w* possible prey types
b_{ik} = the total biomass of prey type *i* in the kth scat, where there are *w* possible prey types
s = total number of fecal samples containing prey

Overall dietary contribution was also calculated using percent modified frequency of occurrence (MFO), and the test index percent Split-Sample Frequency of Occurrence (SSFO) methods (Olesiuk et al. 1990). An assessment of scat resampling error levels was made using bootstrapping techniques in which median (500th sorted point estimate) diet estimates and nonparametric 95% confidence intervals (25th and 975th sorted point estimates) were derived by randomly resampling scats with replacement and bootstrapping 1000 times.

In order to assess the limitations of different dietary indices to scat field sampling protocols we used bootstrap resampling with replacement to sample 20, 30, 40, 50, 70, 100, 150 scats randomly from the total number of scats available and repeated the process 1000 times. An assessment of scat sample size error levels was described using BR-Fixed and DNA-fixed median (500th sorted point estimate or 50%ile) diet estimates and nonparametric 95% confidence intervals (25th and 975th sorted point estimates).

DNA-based prey detections and relative proportions were produced via custom bioinformatics routines and provided by Chad Nordstrom (Coastal Ocean Research Institute) following high-throughput sequencing performed at the Molecular Genetics Laboratory, Pacific Biological Station, Nanaimo. DNA sequence data was used to determine the contribution of prey species within each scat using "relative read abundance" as described in Deagle et al. (2018). DNA sequence data can provide an estimate of prey proportion within each scat which allows one to calculate a DNA-Fixed diet estimate, but not a DNA-Variable estimate. Hard part information was used to determine salmon size class information for those scats containing salmon.

Table 1 Prey size estimation information. FL=fork length, SL=standard length, TL=Total length. DFO = Fisheries and Oceans Canada, Unpubl. Data. Length-Weight allometric regressions follow: Weight = $a(\text{prey Length})^b$

Species	Source of data, date and location	Length, cm	A	b	R^2	n	Estimated weights (g)
Pacific Herring (Clupea pallasii)	DFO 2015 & 2016, Strait of Georgia	FL	0.0161	2.9472	0.8885	11301	
Pacific Hake (Merluccius productus)	DFO 2015, Strait of Georgia	FL	0.0057	3.03	0.9928	1688	
Walleye Pollock (Gadus chalcogrammus)	DFO 2015, Strait of Georgia	FL	0.0055	3.0824	0.9919	642	
Lingcod (Ophiodon elongatus)	DFO 2015, Strait of Georgia	FL	0.0023	3.3338	0.9993	79	
Plainfin Midshipman (Porichthys notatus)	DFO 2015, Strait of Georgia	TL	0.0043	3.2488	0.96	42	
Shiner Perch (Cymatogaster aggregata)	DFO 2015, Strait of Georgia	FL	0.0672	2.3906	0.7782	96	
Norther anchovy (Engraulis mordax)	Gartz 2004, California	SL (mm)	0.0000356	3.1423	NA	315	
Salmon sp. (Coho salmon, Oncorhynchus kisutch)	Harvey 2000, California	SL	0.0103	3.092	0.989	43	
Salmon spp. (Juvenile, multispecies)	DFO 2016, Salish Sea	FL	0.0103	3.0318	0.9866	3154	
Skate (Raja sp., unidentified)	McCully et al. 2012, UK	TL	0.0046	3.082	NA	6196	
Lamprey sp. (Lampetra sp.)	C. Neville, DFO, Pers. Comm., BC	Estimated weight					315
Dogfish (Squalus suckleyi)	C. Neville, DFO, Pers. Comm., BC	Estimated weight					108
River lamprey (Lampetra ayresii)	C. Neville, DFO, Pers. Comm., BC	Estimated weight					24
Squid sp. (unidentified)	C. Neville, DFO, Pers. Comm., Salish Sea	Median weight					14
Cephalopod (medium large to very large sizes)	Hunsicker et al. 2000, Alaska	Mean weight +/- 50% SD					247-576

Table 2. Prey species categories and key prey species 'groups' together with number of prey identifed and Numerical Correction Factors (NCF) by length class (where applicable) applied to prey recovered as hard parts identified in 212 harbor seals scats collected in the Strait of Georgia in 2016. For some species size-specific NCFs are provided. Lower and upper 95% confidence intervals are provided for NCFs, noting values in italics were not derived experimentally.

Prey species category	Prey species 'groups'	Number of prey	NCF length class	NCF	NCF lower 95% CI	NCF upper 95% CI
3SP. STICKLEBACK	Forage fish	17	NA	*2*	*1.8*	*2.2*
ANCHOVY	Forage fish	24	NA	7.25	4.47	19.19
BLACK PRICKLEBACK	Other	1	NA	*2*	*1.8*	*2.2*
CEPHALOPOD	Cephalopods	7	NA	1.3	1.14	1.42
DOGFISH	Other	3	NA	*1*	*0.9*	*1.1*
DOVER SOLE	Flatfish	1	NA	*1*	*0.9*	*1.1*
ENGLISH SOLE	Flatfish	4	NA	*1*	*0.9*	*1.1*
EULACHON	Forage fish	1	NA	7.25	4.47	19.19
GADID	Gadids	1	<20 cm	2.05	1.3	4.9
GADID	Gadids	2	20-34 cm	1.3	1.17	1.43
GOBY SP	Other	3	NA	*2*	*1.8*	*2.2*
GUNNEL	Other	5	NA	*2*	*1.8*	*2.2*
HAKE	Gadids	6	<20 cm	2.05	1.3	4.9
HAKE	Gadids	647	20-34 cm	1.3	1.17	1.43
HAKE	Gadids	20	>34 cm	0.72	0.67	0.78
HERRING	Forage fish	362	NA	1.76	1.49	2.16
IRISH LORD SP	Other	3	NA	1.82	1.42	2.52
LAMPREY SP	Other	2	NA	*1*	*0.9*	*1.1*
LING COD	Hexagrammids	4	>60cm	0.72	0.67	0.78
NORTHERN CLINGFISH	Other	1	NA	*2*	*1.8*	*2.2*
PERCH SP	Forage fish	1	NA	*2*	*1.8*	*2.2*
PLAINFIN MIDSHIPMAN	Other	8	<20 cm	*2*	*1.8*	*2.2*

PLAINFIN MIDSHIPMAN	Other	19	>20 cm	*1*	*0.9*	*1.1*
POLLOCK	Gadids	31	<20 cm	2.05	1.3	4.9
POLLOCK	Gadids	26	20-34 cm	1.3	1.17	1.43
POLLOCK	Gadids	10	>34 cm	0.72	0.67	0.78
RIVER LAMPREY	Other	4	NA	*2*	*1.8*	*2.2*
ROCK SOLE	Flatfish	1	NA	*1*	*0.9*	*1.1*
ROCKFISH SP	Rockfish	6	NA	*1*	*0.9*	*1.1*
SALMON (JUV)	Salmon	15	NA	1.85	0.98	14.56
SALMON (ADULT)	Salmon	8	NA	1.43	1.02	2.43
SAND LANCE	Forage fish	10	NA	5.09	3.66	8.28
SARDINE	Forage fish	2	NA	7.25	4.47	19.19
SHINER PERCH	Forage fish	49	NA	*2*	*1.8*	*2.2*
SHORTFIN EELPOUT	Other	13	NA	*2*	*1.8*	*2.2*
SKATE	Other	1	<20 cm	3.13	2.817	3.443
SKATE	Other	1	>20 cm	1.85	1.665	2.035
SLENDER SOLE	Flatfish	21	NA	*1*	*0.9*	*1.1*
SQUID SP	Cephalopods	23	NA	1.02	1.0	1.05
STAGHORN SCULPIN	Other	2	NA	*1*	*0.9*	*1.1*
TUBESNOUT	Other	1	NA	*2*	*1.8*	*2.2*
UNIDENTIFIED FISH	Other	2	NA	*1*	*0.9*	*1.1*

3. Results

3.1 Dietary reconstruction based on prey hard parts

A total of 204 of 212 scats processed contained 365 prey species hard part occurrences from more than 30 prey species. A total of 1366 individual prey items were detected, numerically dominated by Pacific hake (49.3%) and Pacific herring (26.5%), followed by Walleye pollock (4.9%) and Shiner perch (3.6%)(Table 2). Most of the Pacific hake (96%) were in the size range 20-34 cm. Salmon was detected in 12 scats, 2 of which contained both juvenile and adult sizes. Breaking this down, a total of seven scats contained hard parts from 15 Salmon classified as juveniles (median size 23 cm) and another 8 Salmon classified as adults (median size 34-47 cm), also distributed across seven scats in total. After the application of NCF, all 204 scats represented ~229 kilograms of reconstructed prey biomass (scat mean prey biomass = 1,122g, standard deviation = 1,799g, range 29-17,752g).

The percent diet contribution (and 95% bootstrap resampling confidence intervals) by species group for each major dietary index based on prey reconstruction using hard parts methodology is provided in Tables 3-5 and diet contribution by common species names in Table 7. All diet indices are dominated by Gadids (mainly Pacific hake and to a lesser extent Pollock) and Forage fish (mainly Herring and to lesser extent Shiner perch). The BR-Fixed (with numerical correction factors, NCF) contribution of Salmon is 3.6% (95% CI = 2.0-5.4%), similar to BR-fixed without NCF (Tables 4 and 5, Figure 1-3). Adult Salmon make up ~55% of the total. BR-Variable (with NCF) diet contribution of Salmon is higher at 6.3% (95% CI = 2.5-11.4%) (Figure 1), reflecting the contribution of larger Salmon to the total mass across all prey (Table 6). For this index, adult Salmon contribute ~75% of the reconstructed salmon biomass total (Table 8).

As expected, the application of NCFs reduce the importance of Gadids and increases the importance of Forage fish (Table 8), especially when using the BR-Variable indices. The sensitivity of BR-Variable (both with and without NCFs) to the influence of large prey is well illustrated by the results for the Hexagrammid Ling cod. Four large (length 64-87.5 cm) Ling cod (the only Hexagrammids identified) were detected in four scats. This results in 1.4-2% occurrence, 1.8-2.0% for BR-Fixed (with and without NCFs), but 6.9-13.1% for BR-Variable (with and without NCFs).

Table 3. Percent diet contribution by species group using prey hard parts recovered from scats (median i.e. 50%ile and lower 2.5%ile and upper 97.5%ile confidence intervals based on bootstrap resampling with replacement) for prey occurrence dietary indices (MFO=% modified frequency of occurrence, SSFO= % split sample frequency of occurrence).

Species Group	MFO (median)	MFO (2.5%ile CI)	MFO (97.5%ile CI)	SSFO (median)	SSFO (2.5%ile CI)	SSFO (97.5%ile CI)
**Gadids**	46.9	43.8	50.7	53.0	49.1	57.4
**Forage Fish**	30.6	27.8	33.7	29.3	25.6	32.9

**Other**	8.6	6.3	10.7	7.0	4.9	9.1
**Salmon**	3.7	2.1	5.1	2.8	1.5	3.9
**Cephalopods**	5.5	3.6	7.2	3.7	2.4	4.9
**Hexagrammids**	1.4	0.5	2.0	1.2	0.4	2.0
**Rockfish**	1.0	0.0	1.5	1.1	0.0	1.6
**Flatfish**	2.4	1.0	3.6	1.9	0.8	2.9

Table 4. Percent diet contribution by species group using prey hard parts recovered from scats (median and 2.5%ile and 97.5%ile confidence intervals based on bootstrap resampling with replacement) for prey biomass reconstruction (BR) dietary indices without numerical correction factors applied to hard parts that account for complete prey digestion (Fixed=uses fixed proportions within each scat based on prey biomass reconstruction, Variable=uses prey biomass reconstruction across all scats to determine proportions)

**Species Group**	BR-Fixed (median)	BR-Fixed (2.5%ile CI)	BR-Fixed (97.5%ile CI)	BR-Variable (median)	BR-Variable (2.5%ile CI)	BR-Variable (97.5%ile CI)
**Gadids**	58.4	54.0	63.4	47.8	37.1	59.5
**Forage Fish**	24.4	20.5	28.4	22.2	15.4	30.6
**Other**	7.1	4.7	9.5	5.7	2.6	10.4
**Salmon**	3.6	1.9	5.2	5.6	2.2	10.2
**Cephalopods**	1.3	0.5	2.0	1.9	0.7	3.5
**Hexagrammids**	2.0	0.6	2.9	13.1	1.8	25.7
**Rockfish**	1.3	0.0	2.1	1.4	0.0	4.0
**Flatfish**	2.1	0.6	3.3	1.3	0.3	2.9

Table 5. Percent diet contribution by species group using prey hard parts recovered from scats (median and 2.5%ile and 97.5%ile confidence intervals based on bootstrap resampling with replacement) for prey biomass reconstruction (BR) dietary indices with numerical correction factors applied to hard parts that account for complete prey digestion (Fixed=uses fixed proportions within each scat based on prey biomass reconstruction, Variable=uses prey biomass reconstruction across all scats to determine proportions)

**Species Group**	BR-Fixed (median)	BR-Fixed (2.5%ile CI)	BR-Fixed (97.5%ile CI)	BR-Variable (median)	BR-Variable (2.5%ile CI)	BR-Variable (97.5%ile CI)
**Gadids**	56.8	52.4	61.8	40.4	30.4	52.4
**Forage Fish**	26.6	22.7	30.9	36.1	25.0	49.1
**Other**	6.8	4.5	9.0	5.2	2.5	9.2
**Salmon**	3.7	2.0	5.3	6.3	2.5	11.4
**Cephalopods**	1.4	0.5	2.0	1.8	0.6	3.5
**Hexagrammids**	1.8	0.4	2.7	6.9	0.9	14.8
**Rockfish**	1.2	0.0	2.0	1.0	0.0	3.1
**Flatfish**	1.9	0.5	3.0	1.0	0.2	2.2

3.2. Dietary reconstruction based on DNA

A total of 195 of the same 212 scats contained 373 DNA-based prey species occurrences from 42 prey species, including all 5 species of Pacific salmon (as well as Atlantic salmon). Occurrences were dominated by Pacific hake (n=109), followed by Pacific herring (n=42), then by Shiner perch and salmonids. Walleye pollock and Armhook squid were also notable contributors. Note, salmon hard part data was used to classify salmon detections into juvenile and adult occurrences (as per Thomas et al. 2017). Juvenile salmon were detected across 21 scats (with 26 species occurrences overall which results from different species of Salmon being found in the same scat). Adult Salmon were detected across five scats with 13 species occurrences overall within these five scats.

The percent diet contribution (and 95% bootstrap resampling confidence intervals) by species group for each major dietary index based on DNA methodology is provided in Table 6 and for common species names in Table 8. We provide information for the two general approaches of summarizing DNA sequence count data. Firstly as 'occurrence' (i.e. presence/absence of taxa, MFO and SSFO) and secondly as 'relative read abundance' (i.e. proportional summaries of counts, DNA-Fixed). Similar to hard parts, DNA-Fixed diet contributions are dominated by Gadids (mainly Hake and to a lesser extent Pollock) and Forage fish (mainly Herring and to lesser extent Shiner perch) (Tables 6 and 8). The overall DNA-Fixed contribution of Salmon was also similar to BR-Fixed results at 3.8% (95% CI = 2.0-5.4%), with adult salmon estimated to contribute around one third of this total contribution (Table 8).

Table 6. Percent diet contribution by species group using DNA isolated from scats (median and 2.5%ile and 97.5%ile confidence intervals based on bootstrap resampling with replacement) for prey occurrence dietary indices (MFO=% modified frequency of occurrence, SSFO= % split sample frequency of occurrence) and DNA sequence-based reconstruction using a Fixed reconstruction methodology (Fixed=uses fixed proportions within each scat based on DNA sequencing data).

Species Group	DNA MFO (median)	DNA MFO (2.5% CI)	DNA MFO (97.5% CI)	DNA SSFO (median)	DNAS SFO (2.5% CI)	DNA SSFO (97.5% CI)	DNA-Fixed (median)	DNA-Fixed 2.5% CI)	DNA-Fixed (97.5% CI)
**Gadids**	44.4	41.0	48.0	52.9	48.6	57.1	58.9	54.1	63.4
**Forage Fish**	25.4	22.3	28.1	24.1	20.5	27.6	23.1	19.0	26.9
**Other**	9.1	6.9	10.9	4.5	7.7	5.8	5.9	3.8	7.8
**Salmon**	7.5	5.4	9.6	6.1	4.1	8.2	3.8	2.0	5.4
**Cephalopods**	8.1	6.0	10.1	5.1	3.5	6.6	3.6	1.7	5.2
**Hexagrammids**	2.1	1.0	3.3	2.3	0.6	3.6	2.3	0.7	3.4
**Rockfish**	1.5	0.5	2.5	1.8	0.4	2.7	1.8	0.2	2.7
**Flatfish**	1.9	0.9	3.0	1.6	0.5	2.4	1.2	0.1	1.8

3.3. Dietary reconstruction comparisons

Both methods identified a similar number of prey species occurrences and both methods identified similar contributions from the eight key prey groupings using the Fixed model variant (Figures 1 and 2). Variable BR estimates provided lower contributions of Gadids (both Hake and Pollock) and a higher

contribution of Forage fish (e.g., Herring) (Figures 1 and 3), both largely reflecting the application of NCFs. In addition, Variable BR estimates provide higher contributions of Salmon and Hexagrammids, reflecting the large mass of a small number of individual prey items. Overall, prey group rankings between methods were significantly correlated across both occurrence and reconstruction methods (Table 9) i.e., both methods provided statistically similar prey group rankings.

Occurrence indices were compared across prey species groups based on prey hard part and DNA identifications (Figure 2). The MFO contribution of Salmon exhibited the biggest difference across methods, with two-fold higher occurrence rates for DNA (MFO 7.5%) than hard parts (MFO 3.6%), reflecting both the ability of DNA to achieve salmon species resolution, as well as greater detection rates across 2016 scats. Differences across salmon SSFO were smaller, partly reflecting multiple DNA detections of Salmon species within the same scat. DNA also detected more Cephalopod occurrences.

The contribution of Salmon predicted by DNA-Fixed was 3.8%, very similar as that predicted by BR-Fixed with NCFs at 3.7% or without NCFs at 3.6%. The BR-Variable model predicted 6.3% with NCFs and 5.6% without NCFs, noting that 95% confidence intervals overlap across all estimates and are highest for BR-Variable estimates (reflecting the BR-variable estimates are strongly influenced by a small number of scats).

Confidence intervals were smallest for the more abundant prey groups (Gadids and Forage fish), ranging between ~8-18% of median for both Fixed indices (and 25-35% for BR-Variable) (Tables 5 and 6). For Salmon confidence intervals were larger with 42-47% for Fixed models and 60-81% for BR-Variable. Less abundant prey groups had even wider estimates (Tables 5 and 6).

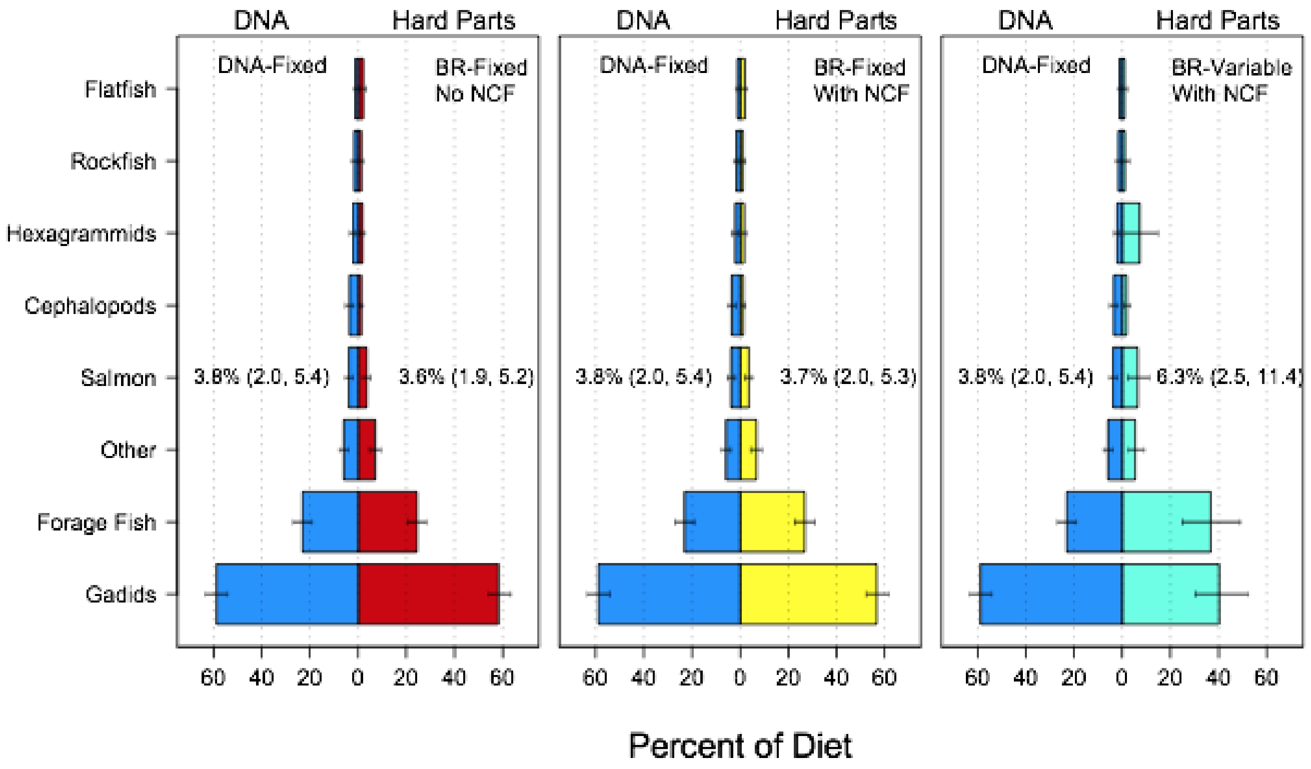

Figure 1 Percent diet contribution by species group comparing diet indices using prey hard parts and prey DNA sequences isolated from scats (median and 2.5%ile and 97.5%ile confidence intervals based on bootstrap resampling with replacement) for DNA-Fixed and Fixed and Variable prey biomass reconstruction (BR) dietary indices without and with numerical correction factors (NCF) applied to hard parts that account for complete prey digestion (Fixed=uses fixed proportions within each scat based on prey biomass reconstruction, Variable=uses prey biomass reconstruction across all scats to determine proportions)

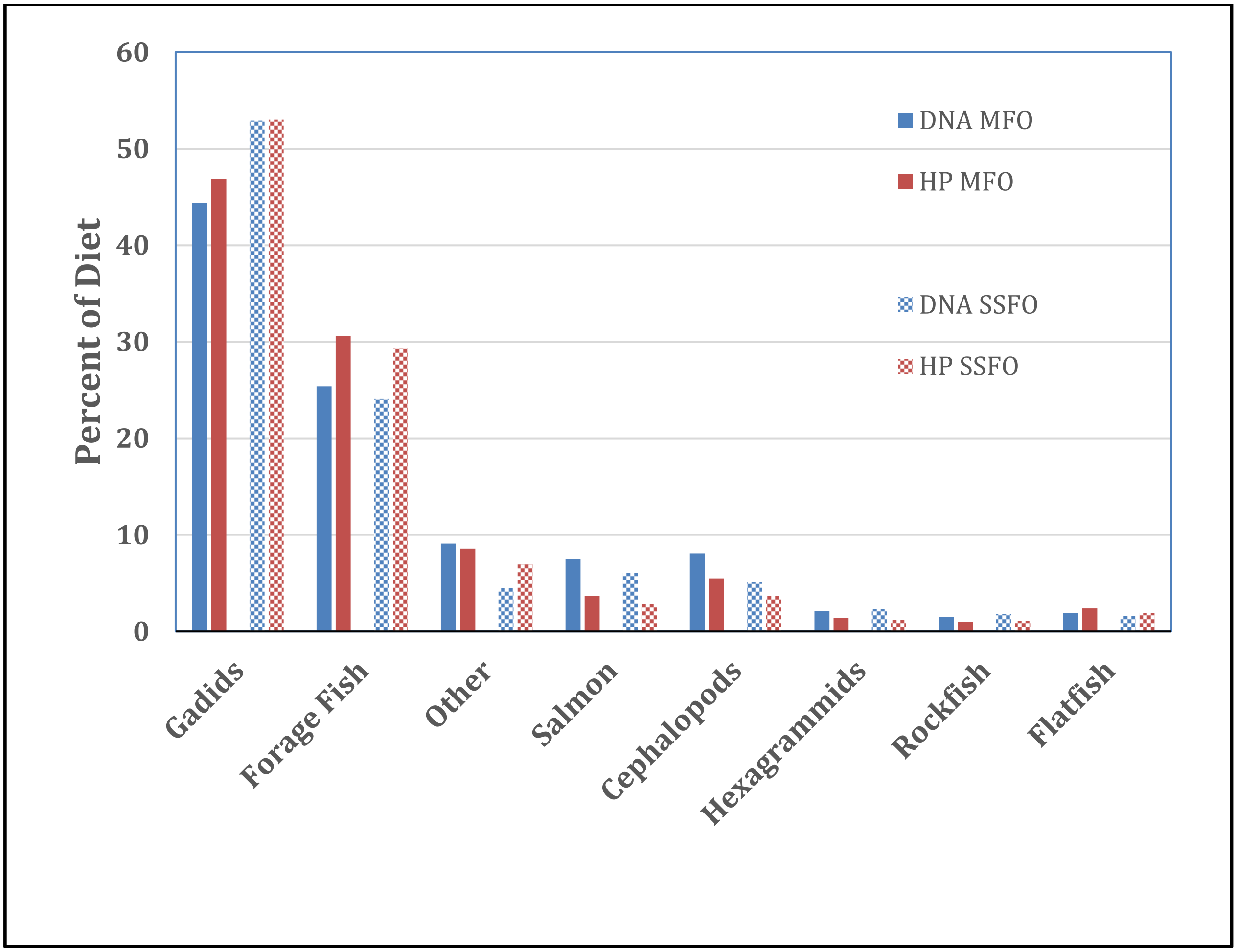

Figure 2 Percent diet contribution by species group comparing occurrence indices using prey hard parts and prey DNA isolated from scats for percent modified frequency of occurrence (MFO) and percent split sample frequency of occurrence (SSFO)

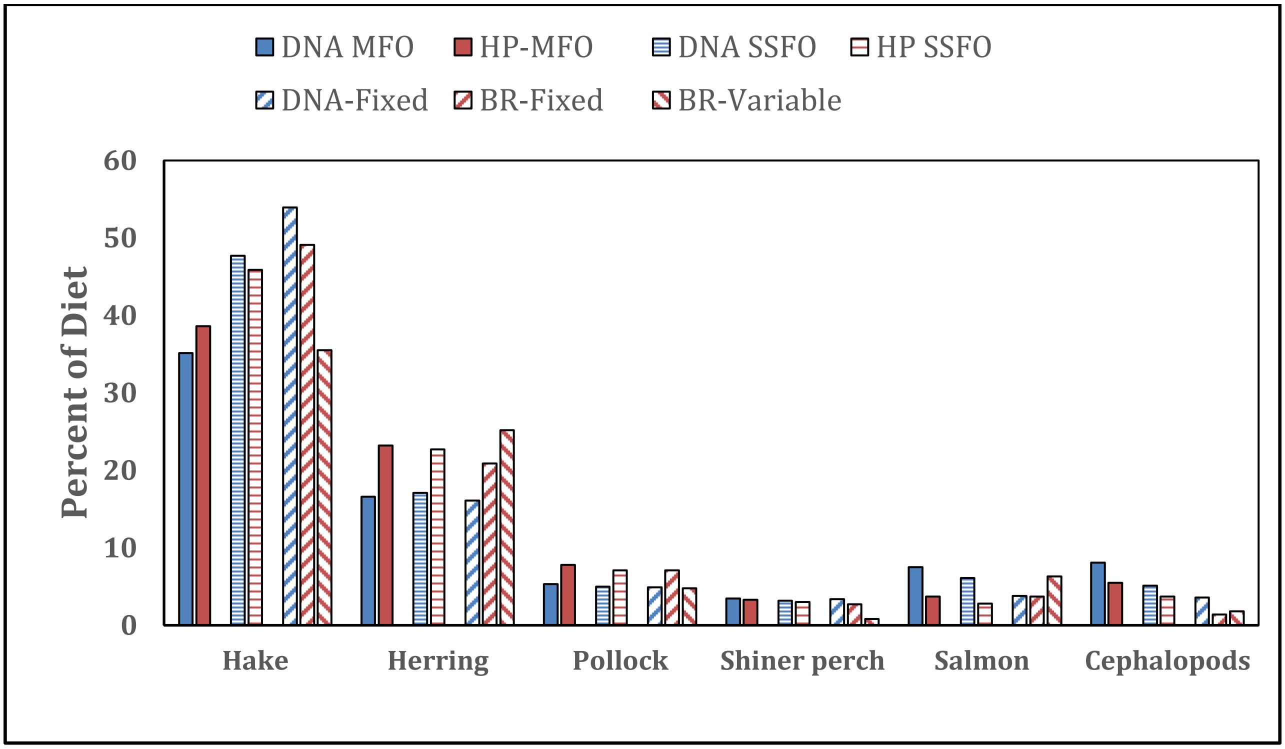

Figure 3. Percent diet contribution by key species using prey hard parts (HP) and prey DNA isolated from scats comparing occurrence indices (percent modified frequency of occurrence (MFO) and percent split sample frequency of occurrence (SSFO)) and DNA-Fixed and Fixed and Variable prey biomass reconstruction (BR) dietary indices with numerical correction factors applied to hard parts that account for complete prey digestion (Fixed=uses fixed proportions within each scat based on prey biomass reconstruction or on DNA sequencing data, Variable=uses prey biomass reconstruction across all scats to determine proportions)

3.4. Effects of scat sub-sampling protocols on dietary reconstruction

A major potential bias in estimating diet from scats is making that assessment with too few scats (termed sampling error). To assess this bias, percent diet contribution by species group for a range of scat sample sizes was compared across comparable diet indices using prey hard parts and prey DNA sequences isolated from scats (median and 2.5%ile and 97.5%ile confidence intervals based on bootstrap resampling with replacement) for DNA-Fixed and BR-Fixed (with NCF) (Figure 4).

This sampling error simulation highlighted a number of clear biases in diet estimation. Firstly, diet estimates diverged from the all scat estimates when sample sizes were small (<50 scats), especially for prey species with low occurrences (e.g., Hexagrammids, Flatfish and Rockfish). With the exception of BR-Fixed forage fish estimates, divergence was found to be an underestimate. Secondly, 95% confidence intervals were as might be expected, far wider when small number of scats were sampled. Typically, 95% confidence intervals approached those based on all scats after 100 scats were sub-sampled. Thirdly, 95% confidence intervals were similar for Gadids, Forage fish and Salmon across methods, but varied across other species groups (e.g. Flatfish).

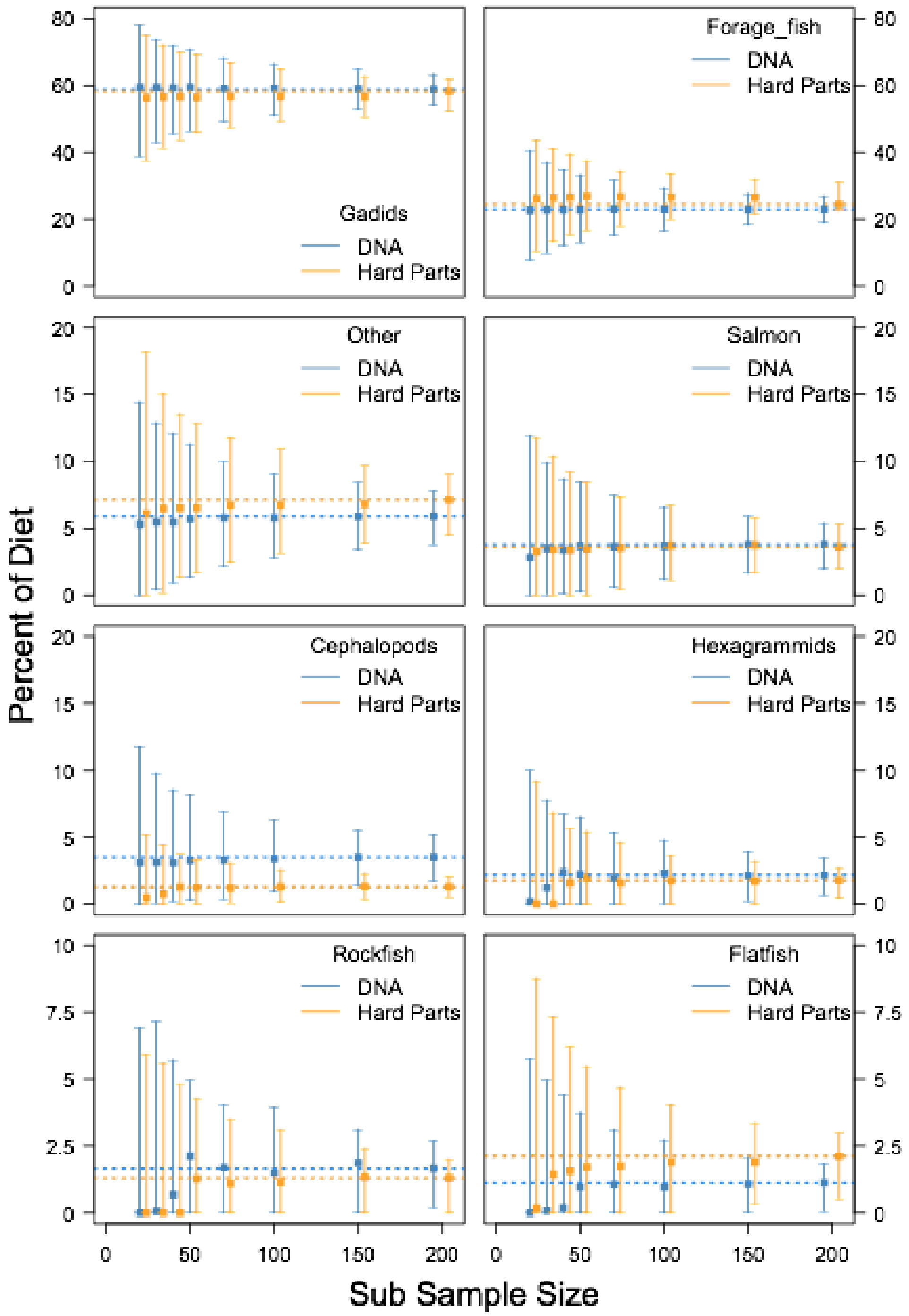

Figure 4 Percent diet contribution by species group for a range of scat sample sizes comparing diet indices using prey hard parts and prey DNA sequences isolated from scats (median and 2.5%ile and 97.5%ile confidence intervals based on bootstrap resampling with replacement) for DNA-Fixed and BR-Fixed with numerical correction factors (NCF) that account for complete prey digestion (Fixed=uses fixed proportions within each scat based on prey biomass reconstruction or on DNA sequencing data)

Table 7. Percent diet contribution by common species name using prey hard parts recovered from scats (the 50%ile or median is shaded with 2.5%ile and 97.5%ile confidence intervals based on bootstrap resampling with replacement) for prey occurrence (MFO and SSFO) and biomass reconstruction (BR) dietary indices without and with numerical correction factors (NCF) that account for complete prey digestion (Fixed=uses fixed proportions within each scat based on prey biomass reconstruction, Variable=uses prey biomass reconstruction across all scats to determine proportions). Salmon diet contributions are highlighted in italics and order based on BR-Fixed with C.F.

**Diet index**	**MFO**			**SSFO**			**BR-FIXED No NCF**			**BR-VARIABLE No NCF**			**BR-FIXED with NCF**			**BR-VARIABLE with NCF**		
**Prey species**	50%	2.5%	97.5%	50%	2.5 %	97.5 %	50%	2.5%	97.5%	50%	2.5 %	97.5 %	50%	2.5 %	97.5 %	50%	2.5 %	97.5 %
**HAKE**	38.6	35.6	41.9	45.9	42.0	50.3	50.6	46.4	55.5	41.6	32.3	53.1	49.1	44.9	54.0	35.5	26.2	46.9
**HERRING**	23.2	20.6	25.9	22.7	19.4	26.1	19.6	15.8	23.3	19.5	12.9	27.5	20.9	17.0	24.6	25.2	17.1	34.7
**POLLOCK**	7.8	5.8	9.5	7.1	4.8	9.0	7.2	4.6	9.5	5.8	2.4	10.3	7.1	4.5	9.3	4.8	2.3	8.1
**SHINER PERCH**	3.3	1.8	4.6	3.0	1.4	4.3	2.5	0.9	3.9	0.6	0.2	1.1	2.7	1.1	4.2	0.8	0.3	1.7
SALMON	*1.9*	*0.9*	*2.9*	*1.5*	*0.6*	*2.3*	*2.1*	*0.7*	*3.2*	*4.5*	*1.6*	*8.1*	*2.0*	*0.8*	*3.1*	*4.7*	*1.6*	*8.5*
**LINGCOD**	1.2	0.4	1.8	1.2	0.4	2.0	2.0	0.6	2.9	13.1	1.8	25.7	1.8	0.4	2.7	6.9	0.9	14.8
**PLAINFIN MIDSHIPMAN**	2.2	0.9	3.2	1.5	0.6	2.3	2.1	0.7	3.3	2.0	0.6	4.1	1.8	0.6	2.7	1.6	0.5	3.2
SALMON (JUV)	*1.9*	*0.9*	*2.8*	*1.3*	*0.5*	*1.9*	*1.5*	*0.5*	*2.4*	*1.2*	*0.3*	*2.7*	*1.7*	*0.6*	*2.6*	*1.6*	*0.4*	*3.5*
**IRISH LORD SP**	0.9	0.0	1.4	1.0	0.0	1.5	1.3	0.0	2.1	0.7	0.0	1.6	1.3	0.0	2.1	1.0	0.0	2.2
**ROCKFISH SP**	0.9	0.0	1.4	1.1	0.0	1.6	1.3	0.0	2.1	1.4	0.0	4.0	1.2	0.0	2.0	1.0	0.0	3.1
**SLENDER SOLE**	1.7	0.5	2.6	1.4	0.3	2.1	1.3	0.3	2.1	0.6	0.1	1.5	1.2	0.3	1.9	0.5	0.1	1.2
**CEPHALOPOD**	1.7	0.8	2.6	1.4	0.6	2.2	1.1	0.3	1.7	1.7	0.5	3.3	1.2	0.4	1.8	1.7	0.5	3.3
**ANCHOVY**	1.2	0.4	1.8	0.7	0.2	1.1	0.7	0.0	1.3	1.6	0.0	5.1	1.1	0.2	1.9	8.4	0.1	23.8
**GUNNEL**	1.2	0.4	1.8	1.1	0.2	1.7	0.9	0.0	1.4	0.3	0.0	0.7	1.0	0.1	1.5	0.4	0.0	1.0
**3SP.STICKLEBACK**	1.3	0.4	2.2	1.6	0.4	2.5	0.9	0.0	1.7	0.0	0.0	0.1	0.9	0.0	1.7	0.1	0.0	0.1
**UNIDENT FISH**	0.4	0.0	0.9	0.8	0.0	1.6	0.8	0.0	1.6	0.1	0.0	0.4	0.8	0.0	1.6	0.1	0.0	0.3
**ENGLISH SOLE**	0.4	0.0	0.5	0.4	0.0	0.4	0.7	0.0	0.8	0.4	0.0	1.2	0.7	0.0	0.8	0.3	0.0	0.9
**GADID**	0.9	0.0	1.4	0.6	0.0	1.0	0.7	0.0	0.9	0.1	0.0	0.2	0.7	0.0	0.9	0.1	0.0	0.2
**DOGFISH**	0.9	0.0	1.4	0.7	0.0	1.1	0.8	0.0	1.1	0.2	0.0	0.5	0.7	0.0	1.0	0.1	0.0	0.3
**SARDINE**	0.4	0.0	0.9	0.4	0.0	0.8	0.3	0.0	0.4	0.1	0.0	0.3	0.6	0.0	0.9	0.6	0.0	1.7

**STAGHORN SCULPIN**	0.4	0.0	0.5	0.1	0.0	0.1	0.7	0.0	0.7	1.6	0.0	5.4	0.5	0.0	0.6	1.2	0.0	4.3
**LAMPREY SP**	0.5	0.0	0.9	0.4	0.0	0.8	0.5	0.0	0.7	0.4	0.0	1.0	0.5	0.0	0.7	0.3	0.0	0.8
**SKATE**	0.5	0.0	0.9	0.3	0.0	0.5	0.3	0.0	0.3	0.2	0.0	0.8	0.4	0.0	0.4	0.3	0.0	1.1
**ROCK SOLE**	0.4	0.0	0.5	0.2	0.0	0.2	0.4	0.0	0.4	0.3	0.0	1.0	0.3	0.0	0.4	0.2	0.0	0.7
**SHORTFIN EELPOUT**	0.4	0.0	0.5	0.2	0.0	0.2	0.2	0.0	0.2	0.0	0.0	0.1	0.2	0.0	0.3	0.1	0.0	0.2
**PERCH SP**	0.4	0.0	0.5	0.2	0.0	0.2	0.2	0.0	0.2	0.1	0.0	0.4	0.2	0.0	0.2	0.2	0.0	0.6
**SQUID UNIDENT**	3.3	1.8	4.5	2.1	1.1	2.9	0.3	0.1	0.4	0.2	0.1	0.4	0.2	0.1	0.3	0.1	0.1	0.3
**SAND LANCE**	0.4	0.0	0.9	0.3	0.0	0.4	0.0	0.0	0.1	0.1	0.0	0.3	0.2	0.0	0.3	0.4	0.0	1.2
**DOVER SOLE**	0.4	0.0	0.5	0.2	0.0	0.2	0.1	0.0	0.2	0.1	0.0	0.3	0.1	0.0	0.1	0.1	0.0	0.3
**GOBY SP**	0.4	0.0	0.5	0.2	0.0	0.2	0.1	0.0	0.1	0.0	0.0	0.0	0.1	0.0	0.1	0.0	0.0	0.1
**RIVER LAMPREY**	0.9	0.0	1.3	0.6	0.0	0.9	0.0	0.0	0.1	0.1	0.0	0.1	0.1	0.0	0.1	0.1	0.0	0.2
**BLACK PRICKLEB**	0.4	0.0	0.5	0.4	0.0	0.4	0.0	0.0	0.0	0.0	0.0	0.1	0.0	0.0	0.0	0.0	0.0	0.1
**EULACHON**	0.4	0.0	0.5	0.4	0.0	0.4	0.0	0.0	0.0	0.0	0.0	0.0	0.0	0.0	0.0	0.1	0.0	0.2
**TUBESNOUT**	0.4	0.0	0.5	0.4	0.0	0.4	0.0	0.0	0.0	0.0	0.0	0.0	0.0	0.0	0.0	0.0	0.0	0.0
**NORTHERN CLINGFISH**	0.4	0.0	0.5	0.1	0.0	0.1	0.0	0.0	0.0	0.0	0.0	0.0	0.0	0.0	0.0	0.0	0.0	0.0

Table 8. Percent diet contribution by common species name using DNA sequence data isolated from scats (the 50%ile or median is shaded with 2.5%ile and 97.5%ile confidence intervals based on bootstrap resampling with replacement) for prey occurrence (MFO and SSFO) and proportions based on prey sequences (DNA-Fixed) (Fixed=uses fixed proportions within each scat based on prey biomass reconstruction). Salmon diet contributions are highlighted in italics and order based on DNA-Fixed.

Diet index	MFO			SSFO			DNA-FIXED		
**Prey species**	**50%**	**2.5%**	**97.5%**	**50%**	**2.5%**	**97.5%**	**50%**	**2.5%**	**97.5%**
**Hake**	35.1	31.7	38.7	47.7	43.2	51.7	54.0	49.1	58.5
**Herring**	16.6	14.1	19.2	17.1	13.9	20.2	16.1	12.8	19.5
**Pollock**	5.3	3.7	6.9	5.0	3.3	6.5	4.9	3.1	6.7
**Shiner Perch**	3.5	2.0	4.8	3.2	1.6	4.5	3.4	1.6	5.0
**Giant Octopus**	2.6	1.3	3.8	1.8	0.9	2.7	3.3	1.4	4.9
**Plainfin Midshipman**	3.4	2.1	4.7	2.5	1.2	3.5	2.1	0.7	3.1
**3SP. Stickleback**	2.7	1.3	3.9	2.3	1.0	3.3	1.8	0.5	2.7
**Lingcod**	1.2	0.4	1.7	1.2	0.2	1.8	1.1	0.2	1.8
**Kelp Greenling**	0.4	0.0	0.9	0.8	0.0	1.7	0.8	0.0	1.7
Juv Atlantic Salmon	*0.4*	*0.0*	*0.9*	0.8	0.0	1.3	*0.8*	*0.0*	*0.9*
**Red Irish Lord**	0.4	0.0	0.9	0.4	0.0	0.9	0.8	0.0	1.6
**Blackbelly Eelpout**	0.8	0.0	1.3	0.6	0.0	0.9	0.8	0.0	1.5
**Anchovy**	0.8	0.0	1.3	0.6	0.0	1.1	0.8	0.0	1.5
**English Sole**	0.4	0.0	0.9	0.3	0.0	0.5	0.8	0.0	1.0
**Brown Cat Shark**	1.3	0.4	2.1	0.9	0.3	1.5	0.7	0.0	1.1
**River Lamprey**	1.3	0.4	2.1	0.8	0.2	1.3	0.7	0.0	1.0
**China Rockfish**	0.8	0.0	1.3	0.6	0.0	1.1	0.7	0.0	1.0
**American Shad**	0.4	0.0	0.9	0.4	0.0	0.7	0.6	0.0	0.8
Coho Salmon	*1.3*	*0.4*	*2.1*	*0.8*	*0.2*	*1.2*	*0.6*	*0.2*	*1.0*
Chinook Salmon	*1.2*	*0.4*	*1.7*	0.5	0.1	0.8	*0.6*	*0.0*	*1.1*
**Pug. Sound Rockfish**	0.4	0.0	0.9	0.3	0.0	0.5	0.6	0.0	0.9
Juv Coho Salmon	*1.9*	*0.9*	2.7	1.1	0.5	1.7	*0.6*	*0.1*	*1.0*
**Sandlance**	0.4	0.0	0.9	0.2	0.0	0.3	0.5	0.0	0.8
Juv Chinook Salmon	*1.6*	*0.8*	2.5	0.9	0.3	1.4	*0.5*	*0.0*	*0.7*

**Redstripe Rockfish**	0.4	0.0	0.9	0.3	0.0	0.6	0.5	0.0	0.5
**Juv Chum Salmon**	0.8	0.0	1.3	0.3	0.0	0.5	0.5	0.0	*0.8*
**Spiny Dogfish**	0.4	0.0	0.5	0.1	0.0	0.1	0.4	0.0	0.5
**Turbot**	0.4	0.0	0.9	0.3	0.0	0.5	0.4	0.0	0.4
**Slender Sole**	0.4	0.0	0.9	0.4	0.0	0.7	0.4	0.0	0.4
Juv Pink Salmon	*1.2*	*0.4*	*1.8*	*1.1*	*0.3*	*1.5*	*0.3*	*0.0*	*0.4*
**Brown Rockfish**	0.4	0.0	0.9	0.4	0.0	0.6	0.3	0.0	0.4
**Spotted Ratfish**	1.1	0.0	1.7	0.6	0.0	1.0	0.3	0.0	0.3
**Armhook Squid**	3.2	1.8	4.4	2.5	1.3	3.4	0.2	0.1	0.3
**Red Octopus**	0.8	0.0	1.3	0.6	0.0	0.8	0.1	0.0	0.2
**Shortfin Eelpout**	0.4	0.0	0.5	0.2	0.0	0.2	0.1	0.0	0.1
**Staghorn Sculpin**	0.4	0.0	0.5	0.2	0.0	0.2	0.1	0.0	0.1
Chum Salmon	*0.4*	*0.0*	*0.9*	*0.2*	*0.0*	*0.3*	*0.1*	*0.0*	*0.1*
**Pacific Cod**	0.4	0.0	0.5	0.2	0.0	0.2	0.1	0.0	0.1
**Bering Flounder**	0.4	0.0	0.5	0.2	0.0	0.2	0.1	0.0	0.1
Juv Sockeye salmon	*1.0*	*0.4*	*1.7*	*0.7*	*0.1*	*1.1*	*0.1*	*0.0*	*0.1*
**Longnose Skate**	0.4	0.0	0.5	0.2	0.0	0.2	0.0	0.0	0.1
Pink Salmon	*0.4*	*0.0*	*0.5*	*0.3*	*0.0*	*0.3*	*0.0*	*0.0*	*0.0*
**Whitesp. Greenling**	0.4	0.0	0.5	0.2	0.0	0.2	0.0	0.0	0.0
**Pacific Tomcod**	0.4	0.0	0.5	0.2	0.0	0.2	0.0	0.0	0.0
**Penpoint Gunnel**	0.4	0.0	0.5	0.2	0.0	0.2	0.0	0.0	0.0
**Pile Surfperch**	0.4	0.0	0.9	0.3	0.0	0.5	0.0	0.0	0.0
**Starry Flounder**	0.4	0.0	0.5	0.3	0.0	0.3	0.0	0.0	0.0
**Black Rockfish**	0.4	0.0	0.5	0.2	0.0	0.2	0.0	0.0	0.0
**Market Squid**	0.4	0.0	0.5	0.4	0.0	0.4	0.0	0.0	0.0
**Surf Smelt**	0.4	0.0	0.4	0.1	0.0	0.1	0.0	0.0	0.0
**Winter Flounder**	0.4	0.0	0.5	0.3	0.0	0.3	0.0	0.0	0.0
Sockeye Salmon	*0.4*	*0.0*	*0.4*	*0.1*	*0.0*	*0.1*	*0.0*	*0.0*	*0.0*

3.5. Statistical Analyses

Spearman Rank correlations between species group indices highlighted that association between all DNA versus BR comparisons were statistically significant.

Table 9. Results of Spearman Rank correlation tests across DNA and hard part indices

DNA index	Hard part index	Spearman Rank correlation (Rho)	P
MFO	MFO	0.976	<0.001
SSFO	SSFO	0.833	0.01
DNA-Fixed	BR-Fixed (NCF)	0.833	0.01
DNA-Fixed	BR-Variable (NCF)	0.826	0.01
DNA-Fixed	BR-Fixed (no NCF)	0.814	0.01
DNA-Fixed	BR-Variable (no NCF)	0.857	0.006

4. Discussion

This study compared harbour seal prey dietary contributions between diet indices based on methods using prey hard parts recovered in scats and methods using prey identified by DNA sequencing, with a focus on diet estimates derived for salmon. The 'occurrence' diet indices are based solely on presence/absence of a prey species or grouping (MFO and SSFO), while the 'quantitative' indices (Fixed or Variable) determine the proportional contribution based on the size of prey and then biomass reconstruction (when using prey hard parts) or based on 'relative read abundance' of prey sequence data (when using DNA, see Deagle et al. 2018 for details).

Across both 'occurrence' and 'quantitative' diet indices there was a significant correlation in the contribution of key prey species groups (including Salmon) determined using prey hard parts versus DNA (all Rho > 0.8)(Table 9). Both methods identified a similar number of prey species occurrences and estimated a diet dominated (40-59%) by Gadids (mainly Hake and to a lesser extent Pollock) followed (22-36%) by Forage fish (mainly Herring and to lesser extent Shiner perch). Salmon contributed between 2.8-7.5% depending on method and index used. Cephalopods, Hexagrammids and Plainfin Midshipman ("Other" prey group) were additional and typically smaller contributors to the overall diet. The dominance of hake and herring in the diet of harbour seals in the Strait of Georgia was previously reported by Olesiuk et al. (1990), but the diet in 2016 clearly exhibited a higher occurrence of both Pollock and Anchovy.

The method comparison between the occurrence index MFO was best correlated (Rho = 0.976). However, the MFO contribution of Salmon exhibited the biggest difference across methods, with two-fold higher occurrence rates for DNA (MFO 7.5%) than hard parts (MFO 3.6%), reflecting both the ability of DNA to achieve salmon species resolution, as well as greater detection rates across 2016 scats. Differences across Salmon SSFO were smaller, partly reflecting multiple DNA detections of

salmon species within the same scat. DNA methods also detected more cephalopod occurrences. MFO considers overall contribution of a species to be the same irrespective of the number of species within a scat, whereas SSFO makes an equal contribution assumption, whereby the more prey species in a scat the less important each one is overall. SSFO seems to be the more reasonable assumption of the two, but the extent of index differences will depend mainly on diet diversity and feeding patterns.

While both methods identified similar contributions from the eight key prey groupings using the Fixed model variant (Figures 1 and 2), Variable BR estimates provided lower contributions of Gadids (both hake and pollock) and a higher contribution of Forage fish (e.g., Herring) (Figures 1 and 3), largely reflecting the application of NCFs. In addition, Variable BR estimates provide higher contributions of Salmon and Hexagrammids, reflecting the large mass of a small number of individual prey items. For example, the contribution of salmon predicted by DNA-Fixed was 3.8%, very similar as that predicted by BR-Fixed with NCFs at 3.7% or without NCFs at 3.6%. Confidence intervals are ~42-47% around this median value. The BR-Variable model predicted 6.3% with NCFs and 5.6% without NCFs (similar to DNA-SSFO estimates of 6.1%), noting that 95% confidence intervals overlap across all estimates but are highest for BR-Variable estimates (reflecting the BR-Variable estimates are strongly influenced by a small number of scats). Thus, one of the limitations of BR-Variable index is that it can inflate the contribution of particularly large (or abundant) prey found in only a few scats (e.g., Hexagrammids and Salmon). The application of NCFs has a lesser effect on resulting estimates of salmon importance, but has a larger effect on Gadids and Forage fish estimates.

Captive studies to date document biomass models perform better than occurrence models across a range of diet scenarios (Casper et al. 2006; Tollit et al. 2006; Philips and Harvey 2009), with variable models providing marginally better predictions of actual mass fed than fixed models, noting that these studies did not vary meal size appreciably. The variable models attempt to capture the unpredictability of foraging and the apparent pulsed nature of hard part recovery in scats. Thus animals presumably foraging most successfully are given a higher weighting. This assumption may also have a tendency to bias towards larger animals and, as observed in our study, can sometimes inflate the contribution of particularly large or abundant prey found in only a few scats. It is therefore particularly important to generate and consider confidence intervals (using re-sampling methods, e.g., Stenson and Hammill 2004) for biomass models (especially variable-based ones) to provide a measure of error and to allow the impact of any outliers to be critically assessed.

Given the differences observed between fixed and variable methods, further fine-scale foraging studies are needed to determine if meal size actually varies systematically with prey type and availability (justifying variable models). Overall, we recommend deriving diet composition estimates using both variable and fixed methods due to the plausible but largely untested assumption that reconstructed biomass of scats reflects variability in foraging success and meal size (consumption), as well as due to differences in digestion and subsequent deposition, and the probable inclusion of incomplete scat samples in most scat studies.

As observed in previous methodological comparisons (Tollit et al. 2009), prey hard parts found in scats can provide information on prey size and numbers, but identifications of Salmon, Rockfish, and often Flatfish and Cephalopods are not possible to species. In contrast, DNA methods can provide far higher

species resolution, with all 5 species of Pacific Salmon as well as Atlantic salmon detected in processed 2016 scats, as well as multiple species of Flounder, Sole and Rockfish detected. Both reconstruction methods ('occurrence' based or 'relative read abundance') detected prey species undetected by the other technique (Tables 7 and 8). This likely reflects longer passage rates of certain hard parts compared to the soft scat material used in DNA analysis, coupled with differences in detection sensitivity. King et al. (2008) reviewed the pros and cons of different DNA-based approaches to molecular analysis of predation. Major areas of difficulty as well as sensitivity issues include short post-ingestion detection periods and cross-amplification, though good primer design and assay optimization can prevent these problems arising. Thomas et al. (2013) also highlighted from captive feeding studies that diet proportions using DNA sequence data were improved by tissue correction factors and prey-specific corrections based on lipid content. No account of this source of bias has been included within our assessment of confidence intervals.

Numerous captive feeding studies have shown that differential erosion and passage rate of prey items in relation to their size and robustness is one of the major sources of error in the analysis of prey remains from scats (Harvey 1989; Tollit et al. 1997; Bowen 2000; Grellier and Hammond 2006). Our study has attempted to take into account these sources of error (e.g., through the use of NCFs and Pacific IDentifications size estimates). However, we note that the pinniped NCFs used were not available for all species and are highly variable for some species (Table 2), and that it was not always possible to apply robust species-specific size NCFs. In addition, size estimates used in this study were median values. Nevertheless, future BR-based diet studies must continue to address digestion-related biases (Cottrell and Trites 2002; Tollit et al. 2003; Grellier and Hammond 2006).

Notably, we don't have any way to compare the diet indices, regardless of which reconstruction method was employed, for these 212 wild collected scats against the 'true diet' of harbour seals in this region, at this time of year. In this study, 212 scats were collected to reconstruct harbour seal diet, ensuring that minimum sample sizes for reliable reconstruction of primary important prey species were met (Trites and Joy 2005). With larger numbers of samples (i.e., >> 94 scat; Trites and Joy 2005), the study is also protected against sampling error associated with such factors as differences in scat volume (Arim and Naya 2003) and differences in dietary preferences of individual animals. On the other hand, pooling all animals into a single spatial assessment for seals in the Georgia Strait, combines a diversity of collection locations that may hide more regional and temporal consumption trends (Arim and Naya 2003). It is considered critical to collect scat samples that reflect the population in time and space if diet estimates are then used to assess annual consumption of certain prey. In reality, it is not clear that a scat represents a sample of the diet at the fine-scale location at which it was collected. Prey passage rate variability may cause bias – with foraging by animals that are more distant or spend long periods at-sea likely underestimated. Harbour seals diet studies are likely less affected by these issues as they typically forage inshore for relatively short durations.

Diet reconstruction is comprised of what percent diet each of the identified components in the scats constituted the original consumption proportions. Reconstructions are therefore restricted numbers between 0 and 100, with all diet components obligatorily summing to 100%. Thus, with rarer species or species groups, there is a higher probability of missing its occurrence with fewer collected scats

(Figure 4; Hexagrammids, Rockfish, Flatfish), and this results in an automatic compensation to increase the percentage of other consumed species. This source of bias in BR-Fixed reconstructions decreases with sample sizes as shown in Figure 4 in which the dashed line represents the all sample reconstruction relative to reconstructions with smaller numbers of scats (X-axis). Many dietary studies tend to pool prey remains into species groups as we have done. By reducing the number of individual species to 8 species groups can reduce the probability of small sample bias. Overall, the sub-sampling error simulation highlighted a number of clear biases in diet estimation. Firstly, diet estimates diverged from the all scat estimates when sample sizes were small (<50 scats), especially for prey species with low occurrences (e.g., Hexagrammids, Flatfish and Rockfish). With the exception of BR-Fixed forage fish estimates, divergence was found to be an underestimate. Secondly, 95% confidence intervals were as might be expected, far wider when small number of scats were sampled. Typically, 95% confidence intervals approached those based on all scats after 100 scats were sub-sampled. Thirdly, 95% confidence intervals were similar for Gadids, Forage fish and Salmon across methods, but varied across other species groups (e.g. flatfish) (Figure 4).

One of the key goals of this project was to determine the consumption of different Pacific salmon species and age classes. Using hard parts analysis, it is notoriously difficult to distinguish between Pacific salmon species. This limitation is minimized using DNA methodology. On the other hand, with hard part remains it is possible to distinguish between juvenile and adult salmon, and in most cases to reconstruct the original size of the salmon consumed. Using DNA methodology, there is no way to determine the size of the original fish that was consumed. For this reason, many researchers are suggesting a combination of both reconstruction techniques would give the best reconstruction of harbour seal diet, at least where consumption of Salmon is of key importance (Tollit et al. 2009).

Overall, this study provides a number of important conclusions relevant to assessing the biases and limitation in estimating the diet of harbor seals in the Strait of Georgia using various methods.

1) Prey group proportions (including Salmon) based on DNA sequence (relative read abundances) were similar to those derived using prey hard part Biomass Reconstruction.
2) DNA detected higher rates of Salmon (and Cephalopods) than hard parts, but Fixed quantification methods provided near identical contributions (3.8% v 3.7%). BR-Variable quantification provided higher estimates of salmon (6.3%) similar to those estimated using DNA-SSFO (6.1%). DNA methodology estimated the contribution of juvenile salmon to be ~33% of the total, while BR methods estimated this to be 75%.
3) BR-Variable allows for an unequal contribution across scats (an approach that aims to allow for differences in meal size and foraging success). As a consequence, the importance of large or abundant prey is often inflated compared to using a BR-Fixed method. Our study highlight this index has comparatively high confidence intervals caused by a small number of scats having a large influence on diet estimates. Further studies are required to fully understand which index might be more realistic, but the two approaches might be considered upper and lower estimates of prey importance.
4) Confidence intervals generated reflect re-sampling of scats and do not account for additional sources such as that involved in scat field sampling success, prey size and number estimation and errors involved in DNA prey identification and sequence quantification. Confidence

intervals are smaller for more abundant prey groups (Gadids and Forage fish), ranging between ~8-18% of the median for both BR- and DNA-Fixed indices. For salmon confidence intervals were larger with 42-47% for both Fixed models and 60-81% for BR-Variable.

5) The application of NCFs to account for complete digestion or loss of hard parts had a relatively small effect on BR estimates of salmon, with effects more apparent on alternate prey groups (Gadids and Forage fish).
6) Our assessment of sub-sampling biases highlights diet estimates diverged (typically underestimated) from the all scat estimates when sample sizes were small (<50 scats), especially for prey species with low occurrences (e.g., Hexagrammids, Flatfish and Rockfish). Confidence intervals were typically large if less than 100 scats were sub-sampled.
7) While this study has shown that the use of DNA sequence data to quantify diet provides estimate similar to that estimated from hard parts, a composite diet estimate using information based on both methods is considered a best case, largely because it provides increased detectability rates, enhanced species identification resolution, and prey size information.

Acknowledgements

We would like to thank PSF for funding this study and DFO for helping with providing unpublished data on local prey length-mass allometric regressions, especially Chrys Neville. Thanks to larger PSF study collaborators Chad Nordstrom, Sheena Majewski, Austen Thomas and Andrew Trites.

References

Arim M, Naya DE. (2003) Pinniped diets inferred from scats: analysis of biases in prey occurrence. Canadian Journal of Zoology. 81(1):67-73.

Bigg, M. A., and I. Fawcett. (1985). Two biases in diet determination of northern fur seals (*Callorhinus ursinus*). Pages 284–291 in J. R. Beddington, R. J. H. Beverton, and D. M. Lavigne, editors. Marine mammals and fisheries. George Allen and Unwin, London, UK.

Bowen, W. D. (2000). Reconstruction of pinniped diets: accounting for complete digestion of otoliths and cephalopod beaks. Canadian Journal of Fisheries and Aquatic Sciences 57:898–905.

Casper, R. M., S. N. Jarman, N. J. Gales, and M. A. Hindell. (2006). Combining DNA and morphological analyses of faecal samples improves insight into trophic interactions: a case study using a generalist predator. Marine Biology 152:815–825.

Casper, R. M., S. N. Jarman, B. W. Deagle, N. J. Gales, and M. A. Hindell. (2007). Detecting prey from DNA in predator scats: a comparison with morphological analysis, using Arctocephalus seals fed a known diet. Journal of Experimental Marine Biology and Ecology 347:144–154.

Cottrell, P.E., and Trites, A.W. (2002). Classifying prey hard part structures recovered from fecal remains of captive Steller sea lions (*Eumetopias jubatus*). Mar. Mamm. Sci. 18(2): 525-539.

Deagle, B. E., D. J. Tollit, S. N. Jarman, M. A. Hindell, A. W. Trites, and N. J. Gales. (2005b). Molecular scatology as a tool to study diet: analysis of prey DNA in scats from captive Steller sea lions. Molecular Ecology 14:1831–1842.

Deagle, D.E. A.C. Thomas, J.C. McInnes, L.J. Clarke, E.J. Vesterinen, E.L. Clare, T.R. Kartzinel, J.P. Eveson (2018) Counting with DNA in metabarcoding studies: how should we convert sequence reads to dietary data? Molecular Ecology https://doi.org/10.1111/mec.14734

Gatz, R (2004) Length-Weight Relationships for 18 Fish Species Common to the San Francisco Estuary. Interagency Ecological Program for the San Francisco Estuary, Vol. 7(2): 49-57

Grellier, K., and Hammond, P.S. (2006). Robust digestion and passage rate estimates for hard parts of grey seal (*Halichoerus grypus*) prey. Can. J. Fish. Aquat. Sci. 63: 1982–1998.

Harvey, J.T. (1989). Assessment of errors associated with harbour seal (*Phoca vitulina*) faecal sampling. J. Zool. (Lond.) 219: 101-111.

Harvey, J.T., T.R. Loughlin, M.A. Perez, and D.S. Oxman. (2000). Relationship between fish size and otolith length for 63 species of fishes from the Eastern North Pacific Ocean. NOAA Tech. Rep. NMFS 150.

Hunsicker M.E., Essington T.E., Aydin K.Y., & Ishida B. (2010). Predatory role of the commander squid *Berryteuthis magister* in the eastern Bering Sea: insights from stable isotopes and food habits. Mar. Ecol. Prog. Ser. 415:91-108.

Jarman, S. N., N. J. Gales, M. Tierney, P. C. Gill, and N. G. Elliott. (2002). A DNA-based method for identification of krill species and its application to analysing the diet of marine vertebrate predators. Molecular Ecology 11:2679–2690.

Jobling, M., and A. Breiby. (1986). The use and abuse of fish otoliths in studies of feeding habits of marine piscivores. Sarsia 71:265–274.

King, R. A., D. S. Read, M. Traugott, and W. O. C. Symondson. (2008). Molecular analysis of predation: a review of best practice for DNA-based approaches. Molecular Ecology 17:947–963.

Murie, D. J., and D. M. Lavigne. (1986). Interpretation of otoliths in stomach content analyses of phocid seals: quantifying fish consumption. Canadian Journal of Zoology 64:1152–1157.

McCully, S.R., Scott, F., & Ellis, J.R. (20 12). Lengths at maturity and conversion factors for skates (*Rajidae*) around the British Isles, with an analysis of data in the literature. ICES J. Mar. Sci. 69: 1812–1822.

Olesiuk, P. F., M. A. Bigg, G. M. Ellis, S. J. Crockford, and R. J. Wigen. (1990). An assessment of the feeding habits of harbour seals (*Phoca vitulina*) in the Strait of Georgia British Columbia based on scat analysis. Canadian Technical Report of Fisheries and Aquatic Sciences 1730.

Phillips, E.M. (2005). Results of a captive feeding study with Pacific harbor seals (*Phoca vitualina richardii*): Implications for scat analysis. M.S. Thesis, Moss Landing Marine Laboratories, San Francisco State University, San Francisco, CA.

Phillips, E.M., and Harvey, J.R. (2009). A captive feeding study with the Pacific harbor seal (*Phoca vitulina richardii*): Implications for scat analysis. Mar. Mamm. Sci. 25(2): 373-391

Pierce, G. J., and P. R. Boyle. (1991). A review of methods for diet analysis in piscivorous marine mammals. Oceanography and Marine Biology 29:409–486.

Reed, J. Z., D. J. Tollit, P. M. Thompson, and W. Amos. (1997). Molecular scatology, the use of molecular analysis to assign species, sex and individual identity to seal faeces. Molecular Ecology 6:225–234.

Stenson, G.B., and Hammill, M.O. 2004. Quantifying uncertainty in estimates of Atlantic cod (*Gadus morhua*) consumption by harp seals (*Phoca groenlandica*). DFO Canadian Science Advisory Research Document 2004/089

Thomas, A.C. S.N. Jarman K.H. Haman, A.W. Trites and B.E. Deagle (2013). Improving accuracy of DNA diet estimates using food tissue control materials and an evaluation of proxies for digestion bias. Molecular Ecology 23(15):3706-3718

Tollit, D.J., M.J. Steward, P.M. Thompson, G.J. Pierce, M.B. Santos and S. Hughes. (1997). Species and size differences in the digestion of otoliths and beaks: implications for estimates of pinniped diet composition. Can. J. Fish. Aquat. Sci. 54: 105-119.

Tollit, D.J., M. Wong, A.J. Winship, D.A.S. Rosen, & A.W. Trites. (2003). Quantifying errors associated with using prey skeletal structures from fecal samples to determine the diet of Steller's sea lion (*Eumetopias jubatus*). Mar. Mamm. Sci. 19(4): 724-744.

Tollit, D. J., S. G. Heaslip, R. L. Barrick, and A. W. Trites. (2007). Impact of diet index selection and the digestion of prey hard parts on determining the diet of the Steller sea lion (*Eumetopias jubatus*). Journal of Zoology 85:1–15.

Tollit, D. J., S. G. Heaslip, B. E. Deagle, S. J. Iverson, R. Joy, D. A. S. Rosen, and A. W. Trites. (2006). Estimating diet composition in sea lions: Which technique to choose? Pages 293–308 in A. W. Trites, S. Atkinson, D. P. DeMaster, L. W. Fritz, T. S. Gelatt, L. D. Rea, and K. Wynne, editors. Sea lions of the world. Alaska Sea Grant College Program, University of Alaska, Fairbanks, Alaska, USA.

Tollit, D.J., A.D. Schulze, A.W. Trites, P. Olesiuk, S.J. Crockford, T. Gelatt, R.R. Ream and K.M. Miller (2009). Development and application of DNA techniques for validating and improving pinniped diet estimates. Ecological Applications 19(4):889-905.

Tollit, D.J., Wong, M.A., & Trites, A.W. (2015). Diet composition of Steller sea lions (*Eumetopias jubatus*) in Frederick Sound, southeast Alaska: a comparison of quantification methods using scats to describe temporal and spatial variabilities. Canadian Journal of Zoology, 93(999), 361-376.

Tollit, D.J., L. Fritz, R. Joy, K. Miller, A. Schulze, J. Thomason, W. Walker, T. Zeppelin, & T.S. Gelatt (2017). Diet of endangered Steller sea lions in the Aleutian Islands: New insights from DNA detections and bio-energetic reconstructions. Can. J. Zoo 95(11): 853-868.

Trites, A.W., Calkins, D.G., Winship, A.J. (2007) Diet of Steller sea lions (*Eumetopias jubatus*) in Southeast Alaska from 1993-1999. Fish. Bull. 105:234-248.

Trites AW, Joy R. (2005). Dietary analysis from fecal samples: how many scats are enough? Journal of Mammalogy. 86(4):704-712.
